# Heat Shock Proteins and HSF1 in Cancer

**DOI:** 10.3389/fonc.2022.860320

**Published:** 2022-03-02

**Authors:** Anna M. Cyran, Anatoly Zhitkovich

**Affiliations:** Legoretta Cancer Center, Department of Pathology and Laboratory Medicine, Brown University, Providence, RI, United States

**Keywords:** cancer, oncology, HSF1, heat shock protein, chaperone, proteotoxic stress

## Abstract

Fitness of cells is dependent on protein homeostasis which is maintained by cooperative activities of protein chaperones and proteolytic machinery. Upon encountering protein-damaging conditions, cells activate the heat-shock response (HSR) which involves HSF1-mediated transcriptional upregulation of a group of chaperones – the heat shock proteins (HSPs). Cancer cells experience high levels of proteotoxic stress due to the production of mutated proteins, aneuploidy-induced excess of components of multiprotein complexes, increased translation rates, and dysregulated metabolism. To cope with this chronic state of proteotoxic stress, cancers almost invariably upregulate major components of HSR, including HSF1 and individual HSPs. Some oncogenic programs show dependence or coupling with a particular HSR factor (such as frequent coamplification of HSF1 and MYC genes). Elevated levels of HSPs and HSF1 are typically associated with drug resistance and poor clinical outcomes in various malignancies. The non-oncogene dependence (“addiction”) on protein quality controls represents a pancancer target in treating human malignancies, offering a potential to enhance efficacy of standard and targeted chemotherapy and immune checkpoint inhibitors. In cancers with specific dependencies, HSR components can serve as alternative targets to poorly druggable oncogenic drivers.

## 1 Introduction

Eukaryotic cells rely on a tightly regulated state of protein homeostasis to carry out their functions. In physiological conditions, proteostasis depends on the cooperation of molecular chaperones, co-chaperones and proteolytic machinery. Proteins with hydrophobic residues and complex 3-dimensional structures require molecular chaperones to acquire their functional forms. Numerous co-chaperones further improve the specificity and selectivity of the process (1, 2). In stress conditions, the survival of cells depends on the activation of an elaborate cytoprotective mechanism known as the heat-shock response (HSR). The HSR has evolved as a rapid mechanism to upregulate selective gene transcription in response to the accumulation of damaged proteins in cells. It utilizes specific molecular chaperones - the heat shock proteins (HSPs) - as effectors to minimize the toxic effects of abnormal proteins. When confronted with hostile environmental and pathophysiological conditions (such as heat, free radicals, nutrient depletion, protein-reactive chemicals), HSPs act to prevent the aggregation of misfolded and malfunctional proteins. They promote protein refolding and/or direct them for degradation by the ubiquitin-proteasome or other proteolytic systems (3). The HSR is driven by heat shock-inducible transcription factors (HSFs) which bind to the promoter regions of the HSPs and dramatically increase their transcription.

The human genome encodes six HSF isoforms, however, only HSF1, 2 and 4 contain confirmed DNA-binding domains (4). To date, three HSFs have been characterized in humans. HSF1 is expressed in all human tissues and acts as a master regulator for rapid and robust responses of cells to proteotoxic stress. In addition to HSPs, HSF1 also affects transcription of a much broader array of genes such as those involved in cell cycle control, protein synthesis, embryonic development, ribosome biogenesis and glucose metabolism (5, 6). Abnormalities in HSF1 are associated with stress sensitivity, aging, neurodegenerative diseases and oncogenesis (5). HSF2 was initially thought to play only a role in development. However, strong evidence has emerged pointing to its complex interactions with HSF1 and its ability to co-regulate HSR (5, 7, 8). HSF3 is solely a murine factor, and HSF4 is involved in lens development (9).

In this work, we review the role of HSF1 and HSPs in protein quality control in physiological conditions and in cancer. We then analyze the functions of HSF1 in cancer biology, links between its expression patterns and key oncogenic pathways, and clinical significance in specific types of cancer. Finally, we discuss why HSF1 is a suitable candidate for anticancer therapy and describe recent attempts of pharmacological targeting and potentially beneficial drug combinations.

## 2 Proteotoxic Stress as a Stress Hallmark of Cancer

A wide array of genetic alterations in cancers results in a set of common features described as hallmarks of cancer. In 2000, Hannan and Weinberg (10) named six classical hallmarks of cancer. Later they expanded this list by adding evasion of immune surveillance and reprogramming of metabolism (11) Luo et al. (12) proposed further characteristics shared across cancer types and contributing to tumor growth and maintenance of the malignant phenotype. These can be collectively described as cancer stress hallmarks and include genomic instability, proteotoxic, mitotic, metabolic and oxidative stress. Targeting of these attributes is a promising therapeutic strategy. Pervasive presence of mutator phenotype and aneuploidy in cancer create proteotoxic stress by producing abnormal proteins and nonstoichiometric amounts of the components of multiprotein complexes (12). Higher translation rates and abnormalities of cancer metabolism further increase the need for protein quality mechanisms. Thus, survival of cancer cells depends on the robust functioning of proteostatic processes which can be described as non-oncogene addiction. HSF1 itself is not tumorigenic (13) but its depletion restricts growth of diverse cancer lines while having little effect on normal cells (6). Dependence on protein quality mechanisms is more ubiquitous in most cancers than dependence on specific oncogenes and proteostasis represents a pan-cancer target for the development of new drugs.

## 3 Heat Shock Proteins

HSPs have become synonymous with molecular chaperones but have a broad range of functions, including folding of newly translated proteins, assembly of protein complexes, protein trafficking, developmental processes and immunomodulation. They are classified according to their molecular masses; the five major groups include HSP110 (HSPH), HSP90 (HSPC), HSP70 (HSPA), HSP60 (HSPD), HSP40 (DNAJ) and small HSPs (HSPB) (14). HSPs can also be divided in accordance with their function in protein quality control into *holdases*, whose function is to bind and hold misfolded proteins to prevent the formation of toxic aggregates and *foldases* that actively assist substrates in assuming their functional conformation. Chaperones make up about 10% of the overall protein mass of immortalized human cells, half of which consists of HSP70 and the more abundant, HSP90. Although HSPs were first identified as proteins expressed in response to thermal stress, it was soon discovered that within each family, 2/3 of them are constitutively expressed, performing housekeeping functions (15). The specificity and selectivity of association with client proteins is modulated by co-chaperones (16).

### 3.1 HSP90

#### 3.1.1 HSP90 Functions

The HSP90 family has five members: inducible cytosolic HSP90AA1 and HSP90AA2, constitutively expressed cytosolic HSP90AB1, mitochondrial TRAP1 and endoplasmic reticulum-localized HSP90B (Grp94). Cytosolic HSP90s are involved in many cellular functions, including protein folding, steroid signaling, DNA repair, protein trafficking, and immunity (17). HSP90 has several hundred client proteins and exerts profound effects on cellular regulatory pathways, with approximately 60% of kinases, 7% transcription factors and 30% ubiquitin ligases requiring its assistance (18). A comprehensive list of HSP90 client proteins is provided by the Picard lab^1^. HSF1 is both a transcriptional activator and client protein of HSP90 (19). HSPs feature different profiles of binding selectivity; HSP70 acts at an early stage of folding, associating with a wide range of protein regions, whereas HSP90 recognizes specific conformations. HSP90 and HSP70 are known to perform independently many of their protein chaperone activities and also to work together in execution of other protein structure-modifying functions (20). HSP90 is a comparatively specialized chaperone. Its substrates generally fall into three categories: (i) proteins with complex conformations in the last stage of the folding process, (ii*)* multiprotein complexes and (iii) proteins bound with their ligands (18). HSP90 activity is fine-tuned by numerous post-translational modifications, most notably phosphorylation and involvement of co-chaperones, such as AHA and p23 (21). Among many signaling pathways it influences are JAK-STAT, PI3K-AKT, BCR-ABL and NF-kB. Their regulatory aberrations have been linked to metastasis, angiogenesis, decreased apoptosis, EMT and cancer progression (22–24). HSP90 also stabilizes mutated forms of the tumor-suppressor p53 (TP53), hampering growth arrest and apoptosis in response to DNA damage (18).

#### 3.1.2 HSP90 in Cancer

**Figure 1:**
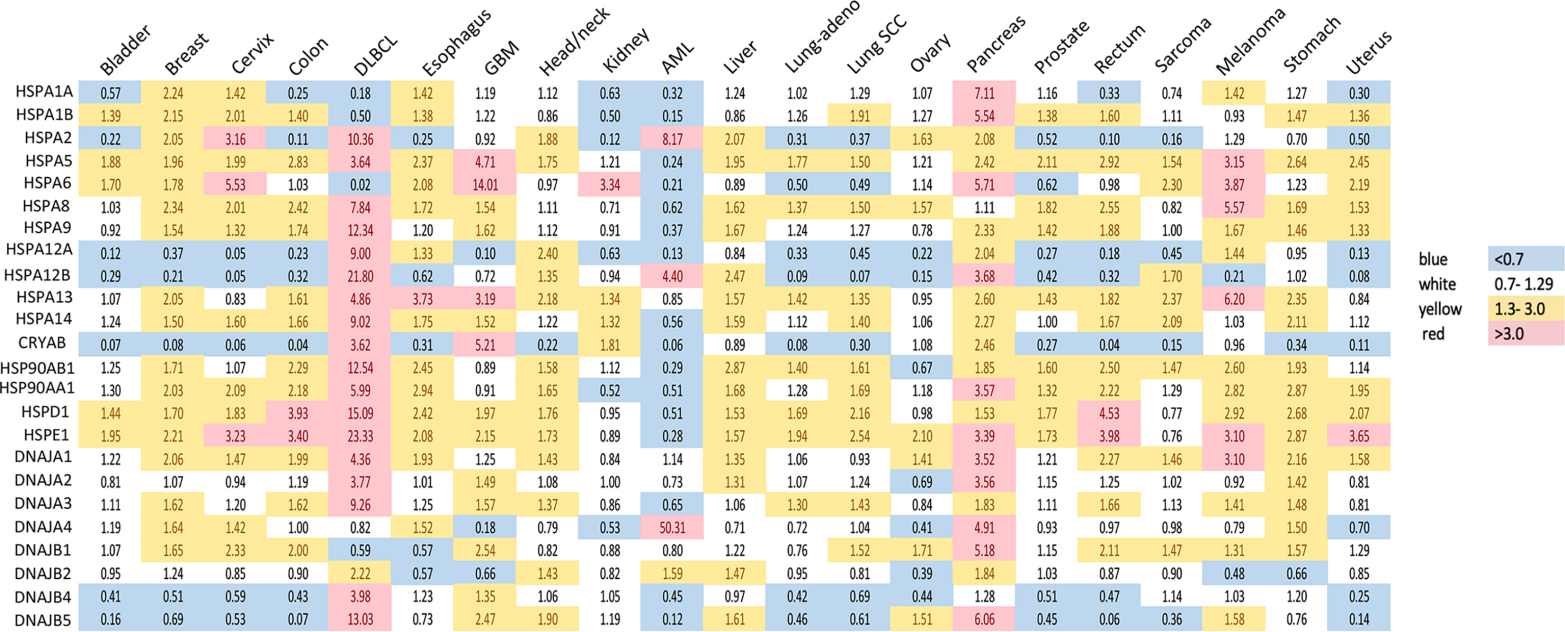
Expression of heat shock genes across different types of human cancers. Shown are fold changes in gene expression of individual HSPs in cancers, which were calculated by dividing the number of transcripts per million in cancer tissue by that in the corresponding normal tissue. Transcript data were accessed through GEPIA (gepia.cancer-pku.cn) (25). Cancer types analyzed: bladder urothelial carcinoma, breast carcinoma, cervical squamous cell carcinoma and endocervical adenocarcinoma, colon adenocarcinoma, diffuse large B-cell lymphoma (DLBCL), esophageal carcinoma, glioblastoma multiforme (GBM), head and neck squamous cell carcinoma, renal clear cell carcinoma, acute myeloid leukemia (AML), hepatocellular carcinoma, lung adenocarcinoma (lung-adeno), lung squamous cell carcinoma (lung-SCC), ovarian serous cystadenocarcinoma, pancreatic adenocarcinoma, prostate adenocarcinoma, rectum adenocarcinoma, sarcoma, skin melanoma, stomach adenocarcinoma and uterine carcinosarcoma. The use of imperfectly matched cell populations as normal controls for hematological malignanies (bone marrow for DLBCL and blood for AML) potentially confounded patterns of normalized HSPs expression in these cancers.

**Figure 2:**
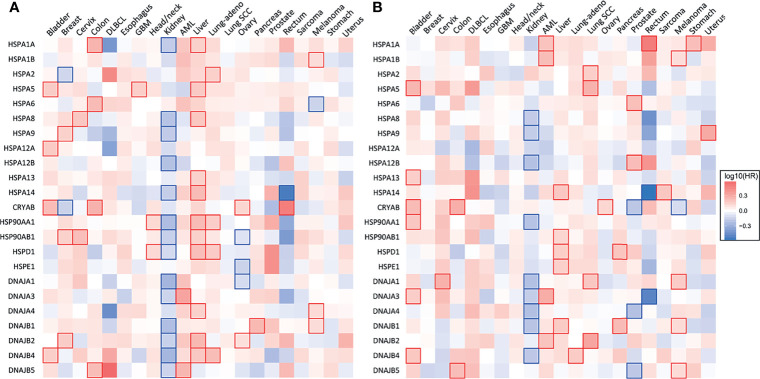
Heat shock gene expression and survival across 21 types of human cancers. **(A)** Heatmap showing the relationship between heat shock gene expression and the overall survival and **(B)** disease-free survival of patients. Patients were divided into groups with high and low mRNA expression at median. Each heatmap cell corresponds to a log_10_ HR (hazard ratio) for respective cancer type. Cells marked with shades of red: HR>0, blue cells: HR<0. Statistically significant relationships are marked with blue 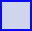 or red 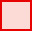 cell borders. Significance level: p<0.05 in log-rank test. Data were accessed through GEPIA (gepia.cancer-pku.cn) (25).

HSP90 has long been recognized as an attractive therapeutic target due to its frequent overexpression in various cancers and its importance in the maintenance of a proper conformation for components of various oncogenic pathways (17, 22). Transcripts of the two most abundant HSP90 members, HSP90AA1 or HSP90AB1, are elevated in 17 out of 21 major human cancers (**Figure 1**). Glioblastoma, renal clear cell (RCC) carcinoma and ovarian carcinoma exhibited approximately normal levels of HSP90 transcripts, whereas acute myeloid leukemia (AML) had lower gene expression of HSP90. High HSP90AA1 or HSP90AB1 expression was associated with a significantly lower survival in patients with breast carcinoma, cervical carcinoma, lung adenocarcinoma and head and neck carcinomas (**Figure 2A**). Overall survival was better for ovarian and RCC cancers with higher HSP90AB1 or both HSP90 forms, respectively. However, high HSP90 expression did not alter disease-free survival of the majority of cancers that showed significantly different overall survival. Only RCC and liver cancers exhibited the same trend for both overall and disease-free survival (**Figure 2B**). Stronger effects of HSP90 expression on the overall survival than progression-free survival suggests that this chaperone may play a more important role in cancer progression/metastasis rather than in the initial therapy resistance. Problems with the accurate determination of the start of disease progression may also hamper analysis of its association with HSP90 and other HSR components.

#### 3.1.3 Targeting HSP90 in Cancer

A multitude of compounds inhibiting HSP90 with promising anticancer properties in preclinical studies was described (26). However, in most cases, their clinical efficacy was modest. Geldanamycin, an ansamycin inhibitor of ATPase activity, showed promise in reducing tumor growth. However, it is poorly soluble and hepatotoxic, which precluded its introduction into clinical practice (27, 28). The synthetic derivative tanespimycin (17-AAG) was proven safe and effective in combination with trastuzumab for the treatment of refractory HER2-positive breast cancer (29). Nevertheless, further studies on prostate, renal, colorectal, head and neck and pancreatic cancers did not confirm the effectiveness of tanespimycin (30–32). The initial success led to the synthesis and testing of other inhibitors of HSP90 with improved bioavailability. For instance, KW-2478, a non-ansamycin, non-purine inhibitor, displayed a good safety profile and some antitumor activity in multiple myeloma *in vivo* with a synergistic action in combination with the proteasome inhibitor bortezomib (33). However, clinical trials with advanced solid tumors (prostate, melanoma, pancreas, gastrointestinal, NSCLC) showed either no significant improvement in survival or high toxicity (32). HSP90 inhibition leads to a compensatory response resulting in the increase of HSP70, which can serve as a biomarker for successful HSP90 blockade (34).

### 3.2 HSP70

#### 3.2.1 HSP70 Functions

The human genome encodes 17 genes and 30 pseudogenes, generating 13 HSP70 gene products, which are subdivided into 7 phylogenetic families. The most abundant and well-studied members are in group VI comprising cytoplasmic and nuclear chaperones (HSPA1, HSPA8, and HSPA6) and group VII encompassing chaperones expressed in the endoplasmic reticulum (HSPA5) (35). Two major inducible forms HSPA1/HSP70-1 and HSPA1B/HSP70-2 differ by two amino acids and are collectively referred to as HSP70. HSPA8/HSP70-8/HSC72 is a constitutively expressed cytoplasmic protein, whereas HSPA6 is inducible and expressed in many types of immune cells (36). HSPA5/HSP70-5/GRP78 is an important facilitator of protein folding and transport across ER membrane (35).

Members of the HSP70 family are present in all cellular compartments and on cell membranes (37). There is some degree of tissue specificity in the expression pattern of HSP70 family members. For instance, HSPA8 and HSPA1A are especially highly expressed in blood vessels and spleen. Yet HSPA1A and HSPA1B, despite the very high degree of homology, are differentially expressed in some tissues (35). HSP70 family members perform crucial housekeeping functions such as nascent protein folding, import of proteins into organelles, protein de-aggregation and assembly of complexes (37). Their simultaneous deletion is lethal (38). Due to a low intrinsic ATPase activity, HSP70 never function alone. Their efficiency and specificity in protein folding are contingent on co-chaperones: HSP40/DNAJ proteins and nucleotide exchange factors (NEF). HSP40/DNAJ proteins are numerous, diverse and assist in the binding of client proteins. Their conserved J domain stimulates ATPase activity, which catalyzes conformational changes. In the next step, NEFs drive ADP and client protein dissociation. The HSP70 protein folding machinery functions in cycles, and its key components can be recycled (39). Proteins with complex structures, such as hormone receptors and transcription factors, undergo a series of folding events and are passed on from HSP40/DNAJ proteins to HSP70 where they are subject to repeated binding and folding. Complex proteins are ultimately delivered to HSP90 (40). NEFs are classified as *(i)* nucleotide releasing factors: HSP-BP-1 type and GRP-type factors; *(ii)* HSP110/HSPH, which are similar to HSP70, in some instances even stress-inducible and capable of preventing protein aggregation independently and *(iii)* less explored BAG family (BAG1-6) (41, 42). In physiological conditions, HSP70 is decidedly more abundant than its co-chaperones, yet their relative concentrations may have a regulatory effect (43).

#### 3.2.2 HSP70 in Cancer

HSP70 transcript levels and their impact on the survival of patients show complex patterns and the extent of expression was not always predictive of the overall or disease-free survival among major human cancers. ER-localized HSPA5 (GRP78) was the most frequently overexpressed HSP70 member whose transcript levels were elevated in 18 out of 21 major human cancers (**Figure 1**). Melanoma, glioblastoma and diffuse B-cell lymphoma had the highest levels of GRP78 overexpression. High expression of this chaperone was predictive of poor overall survival only for three cancers: bladder, glioblastoma and liver (**Figure 2A**). Shorter disease-free survival was associated with higher amounts of GRP78 transcripts only for bladder and squamous cell lung carcinoma (**Figure 2B**). Inducible HSP70 forms are expressed in normal cells even in unstressed conditions, but their levels are elevated in many common human cancers such as melanoma and carcinomas of the breast, cervix, stomach, esophagus, prostate, rectum, pancreas and lung (**Figure 1**). The overexpression of both HSP70 forms was particularly high in pancreatic cancer. A significant association between high expression and lower overall survival was found only for melanoma, colon and liver cancers and no effects were detectable in pancreatic cancer (**Figure 2A**). Overall survival of patients with renal clear cell (RCC) carcinoma was better for the high expression group of HSP1A. High expression of inducible HSP70 was predictive of a shorter disease-free survival only for AML, melanoma and stomach and rectal carcinomas (**Figure 2B**), which is consistent with the impact on the overall survival only for melanoma. HSPA2 and HSPA6 showed elevated gene expression in 9 and 10 out of 21 human cancers, respectively (**Figure 1**), which was predictive of a lower overall survival for one cancer for each chaperone. Other members of HSP70 family showed a less frequent overexpression among major human cancers and their significant impact on the overall survival was generally limited to one cancer. The most common change for HSP12A and HSP12B was downregulation. Collectively, major human cancers overexpressed at least one member of the HSP70 family, and this was associated with lower overall survival for 8 cancers. RCC carcinoma was a clear outlier with a significantly better overall and disease-free survival found for patients with higher mRNA levels of HSP70. RCC is a unique type of human cancer caused by the early loss of the VHL E3 ubiquitin ligase and the resulting constitutive activation of the transcription factor HIF2 (44). Thus, despite a frequent overexpression of HSP70 chaperones, which is indicative of beneficial functions in malignant cells, their abundance does not always associate with more aggressive cancers.

Mechanistic studies have shown that HSP70 downregulation impedes cancer cell growth, migration, invasion in various types of cancer, including colorectal, urothelial, hepatocellular carcinoma, renal cell carcinoma, pancreatic and breast cancers (45–50). While the exact mode of action for each of the HSP70 family members in this capacity has not yet been fully dissected, many molecular insights are available. The importance of HSP70 in cancer appears to center on cytoprotection, either through the HSR or by suppressing apoptotic pathways on many levels. HSP70 prevents BAX from translocating to mitochondria and associating with their outer membranes (51). It also indirectly inhibits the JNK/BIM axis (52). In a different study, HSP70 was found to prevent the release of apoptosis inducible factor (AIF) and cytochrome c from mitochondria and cathepsins from lysosomes by stabilizing their membranes (48, 53). The exact mechanism is not entirely clear, but it is possible that the two mechanisms are either complementary or upstream of one another. HSP70 was also found to inhibit the Apo-2L/TRAIL DISC assembly (54) and its knockdown sensitized cells to TRAIL-dependent apoptosis (55). On the other hand, HSP72 blocks the apoptosis-regulating kinase ASK by directly interacting with it (56). High constitutive transcription of HSP72 in cancer cells also impedes senescence pathways (57). HSP70 is important in tumorigenesis. This is illustrated well by a study using mice injected with cancer xenografts: overexpression of Her2 resulted in malignant transformation only when HSP72 expression was intact. HSP72 depletion inhibited transformation (58). Survival of both normal and malignant cells hinges on the presence of the constitutively expressed HSC70. Its depletion causes G2/M arrest. Knockdown of HSP70-2, the inducible chaperone, predominantly overexpressed in cancers, resulted in G1 arrest (49). There are many other reports detailing functional interactions between cancer hallmarks and HSP70 expression (59). Many substances blocking HSP70 activity have shown promise in human cancer cell lines and xenograft models (60, 61). However, no HSP70 inhibitors are currently in clinical trials, but attempts have been made to repurpose existing drugs with HSP70-inhibiting properties for new indications. This is the case with MKT-077, which exerts anticancer effects by binding to HSPA9 (mortalin). It was tested in phase 1 clinical trials on solid tumors but failed due to renal toxicity and unfavorable pharmacokinetics (62). Another example is methylene blue which showed suppression of HSPA1A and HSPB1 induction and sensitized melanoma cells to other chemotherapeutics (63). Ongoing clinical trials explore the diagnostic potential of HSP70 for the isolation of circulating tumor cells (ClinicalTrials.gov Identifier: NCT04628806) and in prevention where HSP70 DNA is a component of a dual vaccine for the treatment of lesions with increased risk of cervical cancer (ClinicalTrials.gov Identifier: NCT03911076).

### 3.3 HSP40

#### 3.3.1 HSP40 Functions

The largest chaperone family, including over 40 members and 49 genes, some of which encoding several splice variants, is HSP40 (DNAJ). Their key role is partnering with HSP70 and HSP90 and participating in tasks related to the maintenance of proteostasis (64). HSP40 are present in all cellular compartments and partake in protein shuttling, endo- and exocytosis and hormone signaling. Diverse proteins are classified as DNAJ members based on the presence of a conserved J domain, whose role is to enable interaction with other chaperones, stimulate their ATPase and stabilize the client protein binding (65). Based on further structural characteristics, DNAJ are subdivided into 3 categories: DNAJA, DNAJB and DNAJC (66). The classification has functional consequences: DNAJA and DNAJB can bind to aberrant polypeptides independent of ATP to prevent their aggregation, the polypeptide chains they recognize are similar but distinct. An important functional difference is that DNAJA can work independently, whereas DNAJB necessitate HSP70 to prevent aggregation. DNAJC members have a specialized domain that recognizes distinct substrates and delivers them to different HSP70 members, making these highly versatile chaperones more specialized in certain contexts.

#### 3.3.2 HSP40 in Cancer

Gene expression of HSP40 chaperones was elevated in 18 out of 21 major human cancers with particularly high levels of overexpression being found in diffuse B-cell lymphoma and pancreatic cancer, which represents a general trend for HSP expression in these two cancers (**Figure 1**). Bladder, prostate and RCC carcinomas did not show increases in the abundance of any of the HSP40 transcripts. High expression of specific HSP40 forms was associated with lower overall survival in 9 out of 21 major human cancers (**Figure 2A**). A poor survival prognosis in AML, liver carcinoma and melanoma was observed for more than one member of the HSP40 family. High expression of HSP40 was also predictive of shorter progression-free survival in 9 cancers, 8 of which were the same cancers that showed shorter overall survival. As discussed above, this consistency between progression-free and overall survival was not observed for HSP70 and HSP90. As with other chaperones, RCC carcinoma was again a clear outlier that showed a much better overall and progression-free survival in patients with high expression of several HSP40 forms (**Figures 2A, B**). Interestingly, the overall survival of patients with ovarian carcinoma showed opposite trends for DNAJA1 and DNAJB2 expression, pointing to their involvement in different oncogenic programs in this malignancy.

On a protein level, HSP40 expression correlated with a less aggressive cancer phenotype in some studies. DNAJB4 abundance was associated with a better overall survival and lower recurrence rates in NSCLC (67–69). *In vitro*, it inhibited cell proliferation, motility, invasion and slowed cell cycle through STAT1/CDKN1A (70). Other studies in lung cancer provided evidence that overexpression of DNAJB4, mediated by the transcription factor YY1, hinders invasion *via* E-cadherin (71). Conversely, a low expression of DNAJB4 is a feature of highly malignant and metastatic breast and colon cancers (72). DNAJB4 suppresses activity of Src and the formation of oncogenic complexes, such as EGFR-Src, STAT3-Src and FAK-Src (73). It is known that mRNA levels do not always correlate with cellular protein levels (74). While there is a close correlation between the two for HSP90, HSPA5, HSPA8 and HSPB1, it is less clear for other HSP members. The relationship between mRNA and protein abundance varies greatly between tissues; one of the lowest correlation scores is seen in lung. In many cancer types, such as pancreatic, colonic, ovarian and urothelial, the transcript-protein correlation is much lower than in normal tissue (75).

### 3.4 HSP60

#### 3.4.1 HSP60 Functions

HSP60 (HSPD) facilitates the folding, unfolding and degradation of mitochondrial proteins. While performing these classical functions, it is assisted by the co-chaperone HSP10/HSPE. HSP60 forms a double ring of seven subunits (a 14-mer complex) bound to a 7-unit ring formed by the co-chaperone. Together they create a compartment within which client proteins assume their conformation (76). A third of the HSP60 supply is located outside the mitochondria: in the cytosol, on the cell membrane and is secreted in extracellular vesicles (77) broadening its functions to include (among others) protein transport, peptide hormone signaling (78, 79). HSP60 also plays a role in both innate and adaptive immunity and the pathogenesis of autoimmune disorders (80). It induces cytokine release from lymphocytes, dendritic cells and macrophages and interacts with Toll-like receptors 2 and 4, fostering inflammatory response (80, 81).

#### 3.4.2 HSP60 in Cancer

HSP60 (HSPD) transcript was found to be overexpressed in 17 out of 21 human cancers with especially high expression seen in diffuse large B-cell lymphoma and cancers of colon and rectum (**Figure 1**). The expression pattern of HSP60 cochaperone HSP10/HSPE showed a nearly identical trend for overexpression in the same cancers (elevated expression in 18 out of 21 cancers). High expression of HSP60 was predictive of a significantly lower survival in patients with head and neck cancers, liver carcinoma and lung adenocarcinoma (**Figure 2A**). Shorter progression-free survival was found in HSP60 high expression groups of patients with liver and pancreatic carcinomas (**Figure 2B**). As with all other families of HSP proteins, the overall survival of RCC patients was better in the HSP60-overexpressing group. Despite the presence of elevated transcript levels for HSP60 and its cochaperone in the same cancers, high expression of HSP10 was not associated with shorter overall survival in any cancers and in ovarian carcinoma, it was associated with better overall survival (**Figure 2A**). Thus, it appears that cochaperone-independent functions of HSP60 could be more important for disease aggressiveness in patients with head and neck cancers, liver carcinoma and lung adenocarcinoma. As seen with other HSP proteins, HSP60 expression had a more limited prediction potential for progression-free survival than for overall survival.

A multitude of HSP60 functions in cancer and their contributions to the progression and maintenance of specific malignant properties has been established in several cancer models. Mechanistic studies on liver (82), prostate (83), gastric (84), bladder (85), ovarian (86), pancreatic cancers (87), neuroblastoma (88) have shown its association with increased metastatic potential, risk of progression or recurrence and poor overall outcome. HSP60 was highly expressed in Hodgkin lymphoma as well as mitogen-stimulated B-, T- and NK-cells (80, 89). In colonic epithelium, HSP60 is highly expressed during early carcinogenesis and in preneoplastic lesions, suggesting a role in cancer progression (90). HSP60 interacts with major oncogenic pathways, leading to uncontrolled proliferation, resistance to cell death and senescence, and metastatic spread. Its transcription is directly activated by c-MYC, triggering malignant transformation (91). HSP60 is also important for the activity of multiple apoptosis-controlling processes. It promotes activation of IKK-NF-kB pathway, which is crucial for cancer cell survival (92). In pancreatic cancer, HSP60 depletion provokes a decrease in ERK1/2 phosphorylation, leading to cell arrest and apoptosis, ultimately decreasing tumor growth (87). HSP60 also regulates autocrine production of IL-8 *in vitro* and *in vivo via* its upstream regulator TGFβ, mediating resistance to apoptosis (93). HSP60 forms complexes with survivin (a potent inhibitor of apoptosis), stabilizing it and promoting oncogenesis. In experimental settings, HSP60 knockdown decreased the expression of survivin, disrupted mitochondrial homeostasis and led to BAX-mediated apoptosis. In the same study, HSP60 ablation released p53 from its complexes with the chaperone protein restraining its activity (94). Cancer-associated HSP60 overexpression was a prerequisite for a loss of replicative senescence, as p53 was sequestered from interacting with promoters of cell cycle arrest genes (95). A study by Chandra et al. (96) found that a cytosolic accumulation of HSP60 was a common feature of all cells in an early phase of apoptosis induction. The cytosolic accumulation of HSP60 was either of mitochondrial origin, in which case, upon release it interacts with procaspase 3, promoting its maturation and activation. In this model, the chaperone was not available to counteract oxidative stress in the mitochondrion, rendering the cell particularly susceptible to apoptosis. In contrast, HSP60 accumulation of non-mitochondrial origin plays a cytoprotective role (96). As the primary role of HSP60 is to mitigate the effects of reactive oxygen species in mitochondria, its reduction and increased oxidative stress activate AMPK, which inhibits mTOR curbing cell proliferation (86, 97). In glioblastoma, this was accompanied by activation of integrin and WNT pathways and an elevation of EMT markers - key players in the process of metastatic spread (97). HSP60 also promoted metastasis by interacting with β-catenin and α3β1 integrin (98, 99). Interestingly, the latter can be prevented by mizoribine - a pharmacological inhibitor of HSP60 (99).

In some contexts, HSP60 can also assume an anticancer role, as evident by a better survival in RCC patients with high expression of this chaperone (**Figure 2A**). Its knockdown fostered cancer progression by promoting aerobic glycolysis *via* the AMPK pathway and enhanced EMT due to increased oxidative stress in mitochondria (100). In mice, increased tumor size of HSP60-depleted RCC xenografts was observed (100).

#### 3.4.3 HSP60 as Cancer Biomarker and Drug Target

Because HSP60 is widely upregulated in cancer and is present in detectable quantities in blood serum, it was considered a potential biomarker to aid cancer diagnosis and monitor the disease course. Strong evidence exists to support its application for colonic adenocarcinoma, where a specific immunoassay detected increased HSP60 serum levels in cancer patients compared to healthy controls (90). Moreover, HSP60 was elevated already at an early stage of carcinogenesis - in tubular adenoma- raising hope for a tool for early detection (101). Another study on colonic carcinoma found that serum HSP60 had the same sensitivity as carcinoembryonic antigen and provided additional value by correlating with metastatic disease (102).

In HCC, HSP60 levels correlated with alpha-fetoprotein and differentiation grade and predicted favorable outcomes (103). In prostate cancer, HSP60 levels tracked Gleason score, prostate-specific antigen levels and were associated with the development of hormone-refractory disease, prompting further research into whether HSP60 could interact with the androgen receptor (83). Other potential uses for HSP60 as a biomarker include AML (104), lung adenocarcinoma (105, 106), differentiation between different subtypes of glioma (discussed in more detail by Nakamura et al.) (104). HSP60 was found to elicit a humoral response, and autoantibodies against it can have a diagnostic value in breast cancer, especially in DCIS (107) and osteosarcoma (108).

Several pharmacological modulators of HSP60 were described. Among the most promising is the immuno-suppressant mizoribine, which inhibits association with HSP10 and impedes ATPase activity (109, 110). However, given the molar dosage required to achieve biological effects, the compound necessitates further structural refinement. Epolactaene tertiary butyl ester (ETB) covalently binds to cysteine residues of HSP60, allosterically inhibiting its ATPase activity (111). Recently a known activator of apoptosis in cancer cells – myrtucommulone - was shown to interact directly with HSP60 reducing its activity more efficiently than ETB and inducing the intrinsic apoptotic pathway by interfering with mitochondrial functions (112, 113). Other potential HSP60-targeting drugs include carboranylphenoxyacetanilide, known primarily as HIF1 inhibitor, but its biological effects require further study (114). A detailed review of substances proposed to intervene pharmacologically with HSP60, and their activities can be found elsewhere (104, 115).

### 3.5 Small Heat Shock Proteins

Small heat shock proteins (sHSPs or HSPB) are defined by the presence of the α-crystallin domain. The family consists of 11 members, most notably HSPB1 (HSP27), HSPB4 (α-crystallin A) and HSPB5 (α-crystallin B). Their monomeric molecular masses range between 15-40 kDa; however, they usually exist in oligomeric form or as multimeric complexes of up to 40 units (116). sHSPs quickly bind to a large variety of client proteins to prevent the formation of large aggregates. However, they do not induce conformational changes alone. Instead, the unfolded protein chains are passed on to other chaperones for further processing. In contrast to other HSPs, their function is ATP-independent, i.e., client proteins can be released by association with another chaperone rather than by ATP hydrolysis (117). sHSPs are regulated by phosphorylation by multiple kinases, including MAPK2, MAPK3 and PKD (118, 119). They are highly expressed in colorectal (120), pancreatic, breast, ovarian, esophageal and some mesenchymal cancers (116). High expression of CRYAB (HSPB5) was associated with significantly lower disease-free and overall survival for bladder, colon and ovarian carcinomas (**Figures 2A, B**). HSPB1, by far the most researched sHSP member, is linked to tumor invasion and metastasis, epithelial-mesenchymal transition (EMT), reduced apoptosis, drug resistance. An antisense oligonucleotide inhibitor of HSPB1, apatorsen, was tested in phase II clinical trials in patients with prostate, NSCLC (121), pancreatic (122) and urothelial cancers (123), failing to improve the outcomes in all but one study (124).

## 4 HSF1

### 4.1 Structure and Function

HSF1 exists predominantly in the cytoplasm in a monomeric, inactive state. HSF1 monomer consists of the following functional modules: *(i)* N-terminal DNA-binding domain which can also interact with cofactors and modulate transactivation (4), *(ii)* leucine-zipper domains 1-3 (LZ1-3) collectively described as trimerization domain, facilitate interactions with other HSF monomers, *(iii)* regulatory domain which is subject to elaborate modifications, including phosphorylation, acetylation, SUMOylation, *(iv)* leucine-zipper LZ4, repressing oligomerization (9) and *(v)* activation domain located at the C-terminal side. The DBD and oligomerization domains are highly conserved between HSF1 and HSF2 (125). The classical model of HSF1 activation posits that accumulation of misfolded proteins causes HSP70, HSP90 and likely other chaperones to dissociate from monomeric HSF1 and bind unfolded peptide chains (126, 127). This allows HSF1 to trimerize, translocate to the nucleus and bind to the heat shock elements in promoter regions of its gene targets (128). Trimeric HSF1 is capable of binding to DNA, but trimerization alone is not sufficient to transactivate target genes. HSF1 was shown to become active upon phosphorylation (125, 127). Each of the steps requires the assistance of multiple cofactors and different combinations of those fine-tune the overall HSR (4). Detailed studies of HSF1 structure indicate that elevated temperatures induce structural changes in HSF1, such as the unfolding of the regulatory domain and tighter packing of the trimerization region, resulting in increased trimer formation (129, 130). A ribonucleoprotein complex consisting of eEF1a and a non-coding RNA sequence HSR1 was found to promote HSF1 activation (131).

The DNA-binding sites for HSF1 feature inverted repeats of consensus sequence nGAAn. Multiple heat shock elements (HSE) may exist within one gene promoter, and the number of repeats and spatial orientation of bound sequences may vary (132). Attachment of HSF1 and its cofactors to HSE releases polymerase II elongation complex and enhances gene transcription (133). Deletion of HSF1 allows for basal expression of heat-shock proteins but abrogates the acute response (9). During HSR, HSF1 induces the expression of HSPs, co-chaperones and components of the ubiquitin-proteasome system to remove proteins damaged beyond repair (126). The cell cycle is halted to allow time for repair, while the transcription of genes unrelated to damage repair is globally repressed (134). Although short-term heat shock is well tolerated by *Hsf1*-/- animals, prolonged hyperthermia and additional inhibition of HSP90 are deleterious (135). In *Hsf1*-/- mice, heat shock upregulates ~40% of the genes activated in wild-type littermates (135). Mahat et al. (133) used precision run-on sequencing to look at the genome-wide transcriptional profile of HSF1 following heat shock. The study compared transcriptional response in wild-type mice with *Hsf1*-/- and double *Hsf1*-/- and *Hsf2*-/- gene knockouts and showed the HSR was dynamic and robust with approximately 1500 genes being activated and several thousand repressed. Overall, the proposed new model subdivides genes into *(a)* upregulated throughout the HSR, such as the HSPs, *(b)* genes upregulated transiently (only in the initial phase of HSR), such as certain cytoskeletal components, *(c)* large proportion of genes activated at a later timepoint, possibly by transcriptional factors activated by HSF1 and repressed genes. HSF1 can also apparently exert enhancer effects on some genes by regulating their expression from a distance (133, 136).

### 4.2 Regulation of HSF1

Transcription of HSF1 appears to be stable and transcript abundance may be subject to splicing regulation (137). HSF1 is also capable of augmenting its own protein abundance at the translation level. In stress, JNK phosphorylates mTORC1 at Ser567, inhibiting translation. HSF1 salvages mTORC1 from inactivation by binding JNK (138). Dampening of HSR occurs *via* a negative feedback loop exerted on HSF1 by HSP70, HSC70 and HSP40 (DNAJB1). The three chaperones aid monomerization and dissociation of HSF1 from DNA (139). Another mechanism involves overexpression of HSP70, which shifts the balance between HSF1 kinases and phosphatases (140).

HSF1 is subjected to a plethora of post-translational modifications. Phosphorylation occurs mainly within the regulatory domain on multiple serine and threonine residues and can exert both positive (Ser326, T142, Ser320) and negative regulatory effects (Ser303, Ser307, Ser363, T142) (141–143). The first systematic analysis of phosphorylation sites was carried out by Guettouche et al. (144) and identified 12 novel phosphorylated serine residues, including Ser326, which is considered crucial for HSF1 transcriptional activity. HSF1-targeting kinases include AKT1, members of the MAPK family (MEK1, MK2, p38), PLK, PKA, CK2 and mTORC (145, 146). AKT1 phosphorylates multiple sites and activates HSF1 transcriptional activity by fostering association with other co-factors (Ser326, Thr527, Ser230) and allowing trimerization (Thr142) (146). In metabolic stress, AMPK phosphorylates HSF1 at Ser121, suppressing HSR (147). Generally, serine and threonine residues can be modified by several kinases, and one kinase can modify residues with opposing functions. For example, Ser419 phosphorylation by PLK1 facilitates the nuclear translocation of HSF1 (148). In contrast, HSF1 phosphorylation by PLK1 at Ser216 during mitosis mediates its ubiquitination and degradation. This modification also serves another purpose unrelated to HSR - it enables CDC20 to be released from the complex with HSF1 and associate with CDC27, allowing mitosis to progress (149). The complete landscape of phosphorylation events, their timing and role in HSF1 activation is far from clear. However, an engineered HSF1 with 15 disrupted phosphorylation sites was still able to transactivate its gene targets. What is more, the transactivation properties of the mutant were stronger than that of wild-type protein (150). Hyperphosphorylation may regulate the sensitivity of HSF1 to transduced cues triggering activation. A more recent study suggested that phosphorylation at specific residues has an activating effect, whereas hyperphosphorylation results in dampening of HSR (146). Mathematical modeling and experimental work in budding yeast led to the formulation of a two-factor model of HSF1 activation where the key event is its dissociation from HSP70. Phosphorylation events augment the transcription activity and integrate signals from different cellular pathways (151, 152). Other post-translational modifications of HSF1 include its acetylation and SUMOylation.

Acetylation of lysine residues on HSF1 has a two-fold role. In the absence of stress, acetyltransferase p300 modifies Lys208 and Lys298 of HSF1, suppressing its degradation. In stress conditions, the same enzyme additionally acetylates Lys80, inhibiting the binding of HSF1 to the phosphate backbone of DNA (153). Downregulation of p300 results in decreased HSF1 protein levels, due to degradation (153). SIRT1 counteracts this effect by deacetylating HSF1 and promoting a longer association with target gene promoters (154, 155). This form of regulation appears to play a role in pathologies and could be a target for intervention. SIRT1 is upregulated in many cancers, potentially contributing to a chronic hyperactivation of HSF1 in malignancy (154). A similar mechanism involving p300 and SIRT1 regulates HIF1 - a partner of HSF1 in activation of transcription in hypoxic conditions (156, 157). Also involved in the regulation of HSR are histone deacetylases HDAC7, HDAC9 and HDAC1, having both a positive and negative role. HDAC7 and HDAC9 act by removing the inhibitory acetyl group and increase the robustness of HSR. HDAC1, however, may sterically block the DBD of HSF1, diminishing its DNA binding activity (158). Histone deacetylases are a target for a new class of anticancer drugs, some of which have been approved for the treatment of hematological malignancies (159). Other modes of HSF1 regulation include its SUMOylation (160–162) and a production of its differentially spliced isoforms (137, 163).

## 5 HSF1 in Cancer

**Figure 3:**
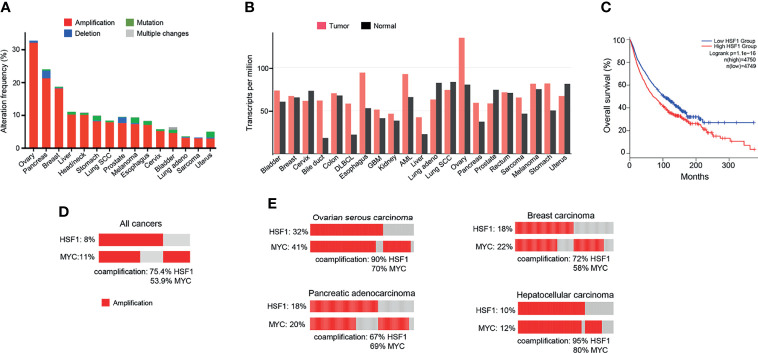
HSF1 alterations in human cancers. **(A)** Frequency of *HSF1* gene alterations in major human cancers. *HSF1* gene is more commonly amplified than mutated in malignancies. Data source: curated set of all studies available (as of January 2022), accessed and analysed through cBioPortal (cbioportal.org) (165, 166). **(B)** HSF1 mRNA levels in cancer and corresponding normal tissue across common cancer types. Data was accessed and analysed through GEPIA (gepia.cancer-pku.cn) (25). **(C)** Kaplan-Meier graph demonstrating the impact of combined alterations in *HSF1* gene on overall survival in a pan-cancer analysis (log-rank p-value=1.1e^-16^). Data were accessed and analysed through GEPIA (gepia.cancer-pku.cn) (25). **(D)** Oncoprint of genetic alterations in *HSF1* and *MYC* genes in a pancancer analysis (n=14107). **(E)** Amplification and coamplification of *HSF1* and *MYC* genes in ovarian serous carcinoma (n=311), invasive breast carcinoma (n=1169), hepatocellular carcinoma (n=596) and pancreatic adenocarcinoma (n=1846) (cbioportal.org) (165, 166).

Since the discovery of HSR, the understanding of the function of the heat shock factors has deepened. It is now clear that HSF1 is involved in many other processes independent of the HSR (164). Unsurprisingly, the cytoprotective properties of HSF1 are exploited by cancers to promote cell survival, proliferation, invasion and metastasis. HSF1 is infrequently mutated across cancer types, but copy number alterations, especially amplifications, are common (**Figure 3A**). The highest frequency of HSF1 gene amplification is found in ovarian, pancreatic, breast and liver carcinomas which also show elevated mRNA levels of HSF1 (**Figure 3B**). Increased HSF1 gene copy number was associated with significantly shorter survival in our pan-cancer analysis (**Figure 3C**). A robust upregulation of HSF1 mRNA in DLBC lymphoma (**Figure 3B**) can at least partially be responsible for strong increases in gene expression for most HSPs in this cancer (**Figure 1**). The cause for a broad decrease of HSPs expression accompanied by a potent induction of only three HSPs (HSPA2, HSPA12B and DNAJA4) in another hematological malignancy AML (**Figure 1**) is less clear as its HSF1 mRNA levels were not reduced (**Figure 3B**). It is possible that the use of peripheral blood cells as a normal tissue control for AML does not provide a good biological reference. In a meta-analysis of 10 studies, including 3159 patients, HSF1 protein abundance was also associated with shorter overall survival in esophageal squamous cell carcinoma, breast cancer, HCC, as well as non-small cell lung cancer (NSCLC) and pancreatic cancer (167). HSF1 expression also correlated with clinicopathological features of aggressiveness such as tumor, nodal and metastasis stage and histological grade (167). Cells with a more malignant phenotype have a higher abundance of HSF1 in the nucleus, and a higher amount of phosphorylated Ser326 (168). Strong nuclear staining for HSF1 was shown in cervix, colon, lung, pancreas, prostate, mesenchymal tumors (168). Association of HSF1 expression with unfavorable prognosis was also shown in oral squamous cell carcinoma (169), gastric (170) and esophageal cancer (171).

HSF1 supports carcinogenesis through a variety of pathways. Upon topical application of a mutagen, *Hsf1*-/- mice developed fewer tumors, had lower tumor burden and survived longer than their wild-type littermates (6). In this model of RAS-driven carcinogenesis, the overall incidence of tumors was much lower when Hsf1 was deactivated, yet once a neoplasm was formed, the proportion of benign and malignant tumors was unchanged. In a separate set of experiments, the impact of Hsf1 deactivation in animals with a dominant p53 mutant was tested. Again, mice with intact Hsf1 function developed larger tumors and survived shortest. *Hsf1*-/+ littermates showed an intermediate phenotype, while the *Hsf1*-/- group was most resistant to carcinogenesis. Heterozygotes developed more carcinomas, while among *Hsf1* wild-type mice, sarcomas were more common. Several common human carcinogens, including a widespread water contaminant arsenic (172) and endogenous/exogenous carcinogen formaldehyde (173) are known to robustly activate HSF1 and HSR, which may play a pro-oncogenic role by promoting survival of damaged cells.

HSF1 fosters cancer cell to grow independently of growth signals (6). Mendillo et al. (168) demonstrated that HSF1 drives a transcriptional program, distinct from the HSR, which supports growth and development of malignant cells. The transcriptional signature involves 456 genes and correlates with poor patient outcomes across a variety of cancers. It encompasses pathways coordinating metabolism, cell cycle, DNA repair, apoptosis, adhesion, translation (168). HSF1 maintains ribosomal biogenesis and promotes glycolysis, which is the main energy-producing metabolic pathway from glucose in cancer cells (6). A bioinformatic analysis of over 10 000 cancer genomes showed that copy number alterations (CNA) of chromosome locus 8q24.3 was a common event across cancers and correlated with poor prognosis (174). Our pan-cancer analysis found that amplifications in HSF1 and MYC genes (both situated at this locus) co-occurred in a high percentage of cases (**Figure 3D**). For the top 4 cancers with the highest frequency of HSF1 amplification (ovarian, pancreatic, breast and liver – **Figure 3A**), the percentage of HSF1 co-amplification with MYC ranged from 67% to 90% (**Figure 3E**). Zhang et al. (175) report that about a third of top 100 most overexpressed genes constituting the HSF1 cancer signature are located at 8q21-24. The authors suggested that the co-expression of the genes cannot be fully explained by their co-localization and raised a possibility of a link to mRNA pre-processing (175).

### 5.1 HSF1 in Major Cancer Types

#### 5.1.1 Breast Cancer

**Figure 4:**
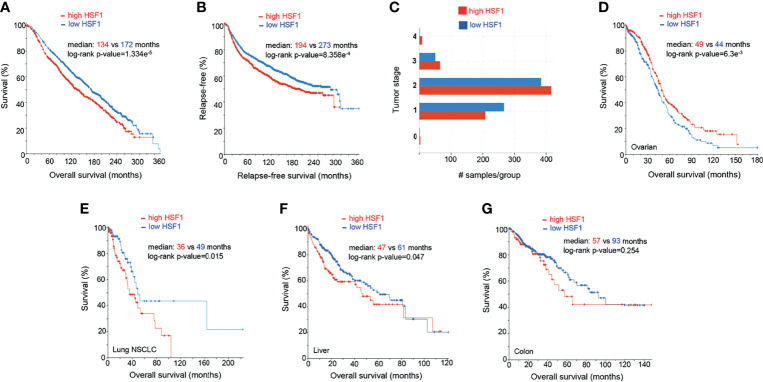
Prognostic significance of HSF1 gene expression in selected human cancers. Patients were divided into groups with high and low expression of mRNA, significance level was defined as p-value<0.05 in log-rank test. Each sample corresponds to an individual patient. **(A)** HSF1 mRNA expression and overall survival in breast adenocarcinoma patients (n=1903). **(B)** Relapse-free survival in breast cancer patients. Cut-off point was set at median expression. **(C)** Tumor stage in relationship to the HSF1 transcript levels. Higher HSF1 expression was associated with more advanced tumor stages, X^2^-test p-value= 1.93e^-3^. **(D)** Overall survival in patients with ovarian serous cystadenocarcinoma (n=537) in relation to HSF1 mRNA expression. Cut-off point was set at median expression. Microarray data used in panels A-D was sourced and analyzed *via* cBioPortal (cbioportal.org) (165, 166). **(E)** Overall survival in non-small cell lung cancer (NSCLC, n=211), **(F)** Hepatocellular carcinoma (n=373), cut-off point was set at 0.5 SD above the mean expression. **(G)** Colorectal adenocarcinoma (n=379), HSF1 mRNA is not associated with survival. RNA Seq data used in panels E-G was sourced and analyzed *via* cBioPortal (cbioportal.org) (165, 166).

In our analysis of publicly available mRNA data (n=1903), high expression of HSF1 in breast adenocarcinoma correlates with poor prognosis (**Figures 4A–C**). Increased levels of HSF1 were associated with shorter overall survival and relapse-free survival. Samples with higher RNA expression of HSF1 also had higher histologic grade (X^2^ test p-value<10^-10^) and tumor stage (X^2^-test p-value= 1.93e^-3^). While most patients with stage I breast cancer had low HSF1 expression, at more advanced stages elevated HSF1expression was more common. Negative ER, PR status and HER2 gain are also more likely in cases with high HSF1 transcripts (X^2^-test p-values: 4.72e^-8^, 1.98^e-10^ and 7.56^e-3^ respectively). While mutations of *HSF1* gene were uncommon, gene amplifications were found in 18% of patients. Previously published meta-analyses of RNA data did not find a statistically significant correlation of HSF1 with prognosis or reported significance for ER-positive patients only, which likely results from a lower number of cases studied (176, 177). Normal breast tissue typically shows no nuclear HSF1 staining, whereas HSF1 was commonly detectable in cancers, most abundantly in high-grade carcinoma (178). The increase in nuclear HSF1 was found at an early stage in carcinogenesis and was already seen in carcinoma *in situ.* Strong nuclear staining of HSF1 correlated with tumor stage and low differentiation (178). Estrogens can promote phosphorylation of HSF1 at S326 in ER-positive breast cancers. The activated HSF1 then fosters the transcription of several critical genes, including HSPB8, LHX4, PRKCE, WWC1, and GREB1 (179). Tumors with elevated HSF1 protein expression were also more likely to be ER-negative, HER2-positive and triple-negative (which typically have a more aggressive course) and were often diagnosed at more advanced stages (178). HSF1 appears to be involved in both ER and HER2 pathways. HSF1 associates with multiple proteins, including HDAC1, HDAC2 and metastasis-associated protein 1 (MTA1). These complexes accumulate in close proximity to promoters of estrogen-responsive genes, inhibiting their expression (180). The development of endocrine resistance involves multiple mechanisms, from overexpression of cytokines to transcription factors (STAT, NFkB, HIF1A, MYC). It is of great clinical interest to resolve the functions of HSF1 and HSPs in this process. HSF1 may decrease transcription and increase the degradation of ERα (181), while HSP70 and HSP90 can bind to steroid receptors and alter their activity (182).

HER2, overexpressed in up to 50% of breast carcinoma cases, is known to promote tumor cell growth. In HER2-positive breast cancer, HSF1 promotes malignant transformation and metastasis. Non-malignant HER2-expressing cells acquire malignant characteristics, manifested as the formation of transformed foci *in vitro* and tumors in mice only in the presence of functional HSF1 (183). HER2 also activates HSF1 and its effectors, including HSP90, which results in the activation of RAS-RAF-ERK1/2 axis. In *Hsf1* ^+/-^ mice, despite overexpression of HER2, tumorigenesis was significantly impaired, which was experimentally observed as decreased proliferation, invasion and EMT (184). Mechanistically, HER2 activated HSF1 *via* the PI3K-AKT-mTOR axis and inhibition of HER2, either pharmacological with lapatinib or transient knockdown, attenuated HSF1 activity (185). Consequently, a feedback loop exists, with HER2 activating HSF1, which in turn activates many of its classical effectors, such as HSP70 and HSP90, which further enhance HSF1 transcription (185). HSF1 and HSPs were consistently and very highly expressed in cells resistant to the tyrosine kinase inhibitor lapatinib, which disrupted both HER2 and EGFR pathways. In this context, targeting HSF1 decreased the expression of ERBB2, mut-p53 and components of several compensatory pathways implicated in resistance to this mode of therapy, suggesting that combination therapy with HSF1 inhibitor could be useful in preventing resistance to lapatinib (186).

#### 5.1.2 Ovarian Cancer

Ovarian cancer is the most lethal female-specific cancer. Serous ovarian adenocarcinoma had elevated HSF1 mRNA expression in comparison to normal ovarian tissue (**Figure 3B**). A higher expression ovarian cancer group had a statistically better overall survival (**Figure 4D**). Gene CNA and chromosomal rearrangements were more prevalent in ovarian cancer than in any other human malignancy (**Figure 3A**). Intrachromosomal rearrangements in ovarian cancers were found to influence gene expression at loci distant from the aberration (187). A genome-wide search identified 8q24 (where HSF1 is situated) as high-risk loci in ovarian cancer (188). However, deletion of 8q24.3 increases 5-year mortality (174). On the protein level, malignant tumors express HSF1 more abundantly than benign ovarian tumors (189). HSF1 was barely detectable by IHC in normal ovarian tissue but high in different types of tumors: serous, mucinous, endometrioid, clear cell (190). Further, phosphorylation of HSF1 at Ser326 in ovarian cancer samples was associated with poor prognosis (191). HSF1 depletion triggered apoptosis in cancerous cells and suppressed carcinogenesis in nude mice implanted with xenografts (190). HSF1 was also linked to EMT in 3D cell culture model of ovarian carcinoma (192). Apart from HSF1 being a potential target of combinatory antitumor therapy, it could also be a useful diagnostic tool. Detection of autoantibodies against HSF1 in early-stage high-grade ovarian serous carcinoma (FIGO Ia-Ic) increased diagnostic accuracy of CA-125 (193). As 81% of ovarian cancers express the ERα (194), resolving the interactions of HSF1 and hormone receptor status could also be clinically important.

#### 5.1.3 Lung Cancer

NSCLC is the most common type of human lung cancer. Higher levels of HSF1 mRNA were associated with lower overall survival in a cohort of NSCLC (**Figure 4E**). HSF1 gene amplifications were found in 11% of these aggressive tumors. On a protein level, a systematic meta-analysis confirmed the inverse relationship between HSF1 and overall survival in NSCLC (167). Moreover, in a study of 105 patients with NSCLC, 42.9% of tissue samples exhibited a high nuclear abundance of HSF1, correlating with poor overall survival, cancer stage and nodal metastasis (195). The same study suggested that nuclear accumulation of HSF1 may play a role in tumor neoangiogenesis (195). Further supporting its importance in malignant properties of NSCLC cells, HSF1 was a key factor allowing them to thrive independently of attachment to a surface. Although depletion of this transcription factor sensitized cancer cells to anoikis, HSF1 activation in normal bronchial epithelium did not confer the ability of anchorage-independent growth (196). HSF1 was also highly expressed in lung adenocarcinoma brain metastases (197). The ABL kinase-HSF1-E2F axis promotes cell cycle progression and cell survival. Its disruption by means of knockdown or targeted pharmacological inhibition provided a proof of concept for a therapeutic intervention (197). HSF1 also directs a malignancy-supporting program in surrounding stromal cells mediated by TGFβ and SD1. Higher expression of HSF1 in stromal cells from patients with stage I NSCLC was associated with significantly shorter disease-free survival (198). HSF1 was also an independent predictor of progression-free survival in NSCLC with positive KRAS and EGFR mutation status (198). A recent study identified HSF1 as a target for pharmacological intervention to overcome the resistance to the EGFR inhibitor erlotinib. The authors demonstrated the effectiveness of this approach using the HSF1 inhibitor emetine to suppress tumor growth in mice (199). Yoon et al. (200) described a natural compound promoting the degradation of the active form of HSF1 through dephosphorylation at S326 in H460 cells, where it induced growth arrest, apoptosis and bolstered the effect of radiotherapy, paclitaxel and cisplatin. In line with the concept of targeting non-oncogene addiction, its effect was more pronounced in cancer cells (200). It would be interesting to see whether this strategy shows similar effectiveness *in vivo*.

#### 5.1.4 Hepatocellular Carcinoma

HSF1 RNA transcripts were more abundant in tumors than neighboring normal liver tissue (**Figure 3B**) and correlated with adverse prognosis (**Figure 4F**). In immunohistochemistry, healthy liver cells showed faint immunoreactivity compared to strong staining seen in HCC cells (201). These observations were corroborated by another study, showing that high protein abundance of HSF1 and phospho-Ser326-HSF1 were increased in HCC and negatively correlated with tumor progression and survival (82). The role of HSF1 in oncogenesis and maintenance was well documented by ablation experiments. HSF1 knockout decreased cell proliferation and upregulated expression of the G1-S inhibitor Rb1 (82). HSF1 knockdown in human HCC cells suppressed cell proliferation and fostered apoptosis by disrupting the PI3K-AKT-mTOR axis (201). The relationship between HSF1 and mTOR, also seen in other cancer types, was demonstrated as a necessary component for MYC-driven HCC (202). Overexpression of cMYC is common in HCC. HSF1 and MYC were amplified in 10 and 12% of samples, respectively and co-occurred for >95% HSF1 cases (**Figure 3E**). Patients with overexpression of both genes had the shortest overall survival and higher-grade tumors. At the same time, human specimen of HCC often features an inactivation of either of the genes; in very rare cases of both (203). HSF1 knockdown downregulated cMYC and slowed the growth of cMYC-dependent HCC in mice. In turn, the silencing of cMYC in human HCC cells downregulated HSF1. In mice with dominant-negative HSF1, the growth of MYC-dependent tumors was completely halted (203). Specific biochemical mechanisms have not yet been demonstrated, but upon HSF1 knockdown, murine tumors overexpressing MYC experienced a dysregulation of its targets involved in metabolism and ribosome biogenesis (203).

#### 5.1.5 Colorectal Cancer

HSF1 is highly expressed in colorectal adenocarcinoma on both transcript and protein level (204, 205). In our analysis, HSF1 RNA expression was not significantly associated with overall survival over 10 years, but patients with elevated HSF1 had lower 5-year survival (**Figure 4G**). HSF1stimulates glutaminolysis *via* GLS1 and activates mTOR in colorectal cancer cells; its knockdown restricts cell growth. Pharmacological inhibition or genetic abolishment of HSF1 suppressed carcinogenesis in mice (205). High expression of HSPs in inflammatory gut diseases and early stages of cancer are well documented (206). A recent study linked HSF1 to colitis-associated colon cancer through inflammatory remodeling of extracellular matrix (206).

#### 5.1.6 Melanoma

HSF1 expression assessed by IHC was associated with greater disease severity as it was stronger in metastatic than in primary lesions. Strong nuclear staining also indicated shorter disease-free survival (207). The upregulation of HSF1 in melanoma likely resulted from its inefficient degradation due to frequent mutations or downregulation of the ubiquitin ligase FBXW7α (207). HSF1 depletion *in vitro* resulted in a reversible decrease in migration and invasion. HSF1 depletion markedly lowered the tumorigenic potential of melanoma cells in nude mice (208).

## 6 Heat-Shock Response and Cancer Immunosurveillance

Tumors grow when the host immune system fails to recognize them as foreign. Cancer cells have evolved different strategies to achieve this, including downregulating the expression of MHC I, eliminating T cells within the tumor tissue and reduced antigen cross-presentation (209). It is well established that chaperones are involved in the immune response through their ability to stably bind polypeptide chains. HSPs bind tumor antigens, forming complexes recognized by monocytes, macrophages, B cells, dendritic cells, ultimately leading to cytotoxic T cell activation. All major HSPs interact with antigen-presenting cells *via* CD40, CD91 and LOX1 (210).

In recent years therapies with PD-1/PD-L1 inhibitors (also known as immune checkpoint inhibitors) have been widely introduced into clinical practice, yielding spectacular success in selected patients, but the overall response rate among all cancers remains around 12% (211). Based on this success, research efforts are invested into gaining a deeper understanding the mechanisms governing their expression and the links to patient outcomes. The binding of PD-1 with its ligand sends an inhibitory signal invoking decreased proliferation and activity of cytotoxic T cells, diminished cytokine production and differentiation of regulatory T cells, allowing cancer cells to escape the host immune surveillance (212). Yang et al. (213) have shown that HSF1 phosphorylation at Thr120 by PIM2 increased its transcriptional activity and promoted binding to HSE in the PD-L1 promoter, enhancing its expression. pThr120-HSF1 was associated with increased migration, invasion and proliferation in breast cancer cells *in vitro*. While targeted inhibition of PIM2 and HSF1 resulted in decreased tumor size in each case, combined treatment had a synergistic effect and arrested tumor growth entirely in murine xenografts (213). Similarly, previous studies show that blocking PIM kinases leads to a decreased PD-L1 expression (214). No studies combining HSF1 inhibitors and PD-L1 inhibitors have yet been published. Therapies targeting multiple HSPs are suggestive of an antitumor potential for a combined HSF1-targeted/immune checkpoint therapy. The effects of a multi-subtype HSP/peptide vaccine tested in murine osteosarcoma were potentiated by the addition of a PD-L1 inhibitor. The combination triggered elevated cytokine production, stimulated CD4+ and CD8+ T cells and mitigated lung metastasis better than each therapeutic modality alone (215). Almost all HSP-inhibitors have been administered so far on intermittent dosage schemes. However, pharmacokinetics seems important, not only for the efficacy of HSF1-HSP axis inhibition but also for the accompanying immune effects. In comparison to a high acute dose regimen, a sustained low-dose inhibition of HSP90 slowed the progression of colon cancer in immunocompetent mice more effectively and produced a higher density of more diverse tumor-associated antigens. In the same experiment, additional administration of an immune adjuvant further improved tumor control and complete ablation of tumor in some cases (216).

HSE is present in the promoters of a plethora of genes, including those related to immune response such as FasL and HLA-G. Hyperthermia elevated the expression of a non-canonical MHC class I molecule HLA-G but did not affect other genes in this class (217). HLA-G is a low-polymorphism gene, generally expressed in tumors and fetal tissues, promoting immune tolerance. It inhibits the proliferation and cytotoxic activity of T cells and NK cells and is known to impair host immune responses against cancer cells (218, 219). Other stress-inducible genes containing HSE in the promoter region include major histocompatibility class I chain-related proteins A and B (MICA and MICB). MICA and MICB bind to NKG2D receptors, activating NK cells. HSF1 knockdown leads to a decrease in MICB only, whereas pharmacological inhibition by NZ28 depressed both MICA and MICB, causing a strong inhibition of NK cytotoxic activity (inhibition of HSP90 did not change MICA and MICB levels) (220). However, the effects of this inhibitor may be partially attributed to its non-specific mode of action and modulation of the NF-kB pathway. The family of NKG2D-ligands comprises six other members (ULBP1-6) whose promoters contain HSE, but their direct activation by HSF1 was not yet established.

## 7 HSF1 and Cancer Therapy

### 7.1 HSF1 and Drug Resistance

Another function of HSF1, independent of the heat shock response, is the promotion of drug resistance. Chemotherapy remains the mainstay of treatment for hematologic malignancies and metastatic cancers, and resistance to cytotoxic agents is common and often leads to therapy failure. One mechanism of multidrug resistance involves a superfamily of membrane transporters - the ATP-binding cassette (ABC) - which pump hydrophobic molecules out of the cell (221). Strong evidence exists that HSF1 contributes to the functioning of an ABC subgroup - ABCB1 (also referred to as P-glycoprotein and MDR1). Elevated P-glycoprotein drastically reduces prognosis in hematologic malignancies, solid and epithelial tumors and can transport a broad range of substrates across cellular membranes such as anthracyclines, taxanes, alkaloids, topoisomerase II inhibitors (221).

MDR1 gene features several HSE regulatory sequences, and its promoter is activated in response to stress (222). The increase in MDR1 expression is not associated with the accumulation of heat shock proteins, and the exact mode(s) of control over MDR1 expression exerted by HSF1 is less clear. Transfection with a constitutively active HSF1 led to increased MDR1 mRNA and protein levels and stimulated vinblastine efflux. The induction of the MDR phenotype was dependent on HSF1 recognizing and binding to binding the HSE in the MDR promoter (223). The concept of HSF1 transcriptional control is supported by the fact that inhibition of HSF1 binding to HSE by quercetin suppressed MDR-dependent drug resistance (224). However, in doxorubicin-resistant osteosarcoma cells, increased HSF1 expression was not coupled to elevated MDR1 transcription as has been shown in a luciferase reporter assay. Also, disruption of the HSE had no effect on induction of the MDR phenotype (225). Melanoma cells overexpressing mutant HSF1 with a deletion in the transcriptional activation domain were resistant to doxorubicin and paclitaxel (but not cisplatin, bortezomib and vinblastine), correlating with overexpression of several ABC transporters (ABCB1, ABCB8, ABCC1, ABCC2, ABCC5 and ABCD1) and increased drug efflux (226). The overabundance of the inactive mutant also suppressed heat shock response through the dominant-negative effect. In contrast, transfection with a constitutively active form of HSF1 elevated HSP expression in the absence of drug resistance (226). Overall, such observations suggest an involvement of HSF1 at a post-transcriptional level. Of note, although ABCs transport a broad array of substrates, the drug efflux induced by HSF1 transfections was selective and differed between cell lines. The reasons for the observed functional specificity remain to be elucidated.

HSF1 is a known regulator of autophagy - a cytoprotective response to stress - which when inhibited, sensitizes cells to radio- and chemotherapy (227). To this end, the transcriptional induction of autophagy *via* autophagy-related protein 7 (ATG7), fosters autophagosome formation and ultimately cell survival (228). Conversely, drug sensitivity was increased following HSF1 knockdown. Consistent with its cytoprotective role, ATG7 expression was associated with poor prognosis in breast cancer patients (229). A few members of the autophagy-related protein family also feature HSE regions, suggesting that these findings can be extrapolated to other proteins, for example, ATG4B, which attenuates the cytotoxicity of epirubicin in hepatocellular carcinoma cells (230). The mechanisms through which HSF1 and autophagy influence drug resistance are likely diverse. An interesting model of autophagy induction was proposed in a study on melanoma cells resistant to apoptosis by ER stress inducers (such as thapsigargin). Stress-induced transcriptional activation of RIPK1 *via* HSF1, downstream of XBP1, consequently promoting cell survival. No HSF1 activation occurred in cells sensitive to apoptosis by ER stress inducers (231).

### 7.2 Proteasome Inhibitors

Multiple myeloma is an example of a disease where a strategy for targeting the malignancy-associated proteotoxic state is successful. Myeloma cells experience elevated proteotoxic stress, not only due to rapid growth and dysbalanced metabolic conditions but also due to rampant immunoglobulin production. This, in turn, leads to unfolded protein response and HSR activation (232). The molecular sequelae of proteasome inhibition involve a stark increase in HSP70, HSP27 and HSF1 expression (233). Silencing HSF1 in myeloma cells, as well as selective pharmacological inhibition of HSF1, strongly sensitizes cells to proteasome inhibition (234). In contrast, downregulation of individual HSPs in combination with proteasome inhibition was not as effective. This treatment was dependent on the basal HSP expression level and HSF1 suppression was less effective in cell lines with already elevated HSPs (235).

### 7.3 HSF1 as a Target for Pharmacotherapy

HSF1 is not an easily druggable molecule as it lacks a discernible binding site for small molecule inhibitors, its activation process is complex, and is subject to numerous posttranslational modifications in response to different forms and degrees of proteotoxic stress. Many potential inhibitors have been developed, often derivates of natural medicinal products, as well as products of *in silico* designs and large-scale screens of synthetic chemical libraries (236). Unfortunately, in many cases, the exact mechanism of action and drug specificity remain unknown. An ideal inhibitor would bind directly and with a high affinity to HSF1. Keeping in mind the potential future use as a clinical drug, the substance should exhibit its function at low concentrations and have favorable pharmacokinetics. To date, only three compounds with a known, on-target mode of action have been identified: KRIBB11, I_HSF1_115, SchA and iaRNA^HSF1^. A recent review by Dong et al. provides a detailed overview of the available compounds, their structure and mode of action (237).

The first direct inhibitor of HSF1, KRIBB11, was identified in a screen of over 6000 molecules (238). A luciferase reporter assay showed a decrease in HSP70 synthesis. Experimental evidence from affinity chromatography confirmed that KRIBB11 binds to HSF1 and abolishes the recruitment of pTEFb, which is necessary for the release of RNA polymerase II and continuation of transcription. KRIBB11 is highly effective with an IC_50_ = 1.2 μM and 10 μM, almost completely preventing the association of pTEFb to the HSP70-promoter. Experiments in nude mice revealed a 47% decrease in tumor volume and depletion of HSP70. This finding indicated a significant antitumor activity with limited general toxicity (238). Following a structure-activity analysis of HSF1 DBD (DNA-binding domain), I_HSF1_115 was developed (239). Interestingly, despite binding to the DBD, it does not inhibit the HSF1 association with the HSE but rather modulates its transcriptional activity. The efficacy of I_HSF1_115 was experimentally validated in several cancer cell lines. The structure targeted by I_HSF1_115 is also present on HSF2. Therefore, it is possible that this inhibitor affects the function of both transcription factors (239). Salamanca et al. (240) utilized a different strategy and developed an RNA aptamer competing with HSE for binding with the DBD of HSF1. The inhibitory aptamer- iaRNA^HSF1^- was evaluated in yeast, Drosophila and human cancer cells, where a reduction in colony-forming ability and an increase in apoptosis were noted. Difficulties with drug delivery *in vivo* currently hamper the application of this approach to pre-clinical studies (240). Recently, Chen et al. (241) reported HIF1-inibiting properties of deoxyschizandrin (also termed shizandrin A) which is a bioactive molecule derived from a Chinese medicinal plant *Schisandra chinensis*. Evidence from surface plasmon resonance and computer modeling suggested that deoxyschizandrin binds to HSF1 directly by inducing conformational changes to the binding site. The compound caused cell cycle arrest and death by apoptosis in human colorectal cancer cells *in vitro*. However, despite its direct binding to HSF1, deoxyschizandrin also appears to alter other functions in cells (241).

## 8 Concluding Remarks

**Figure 5:**
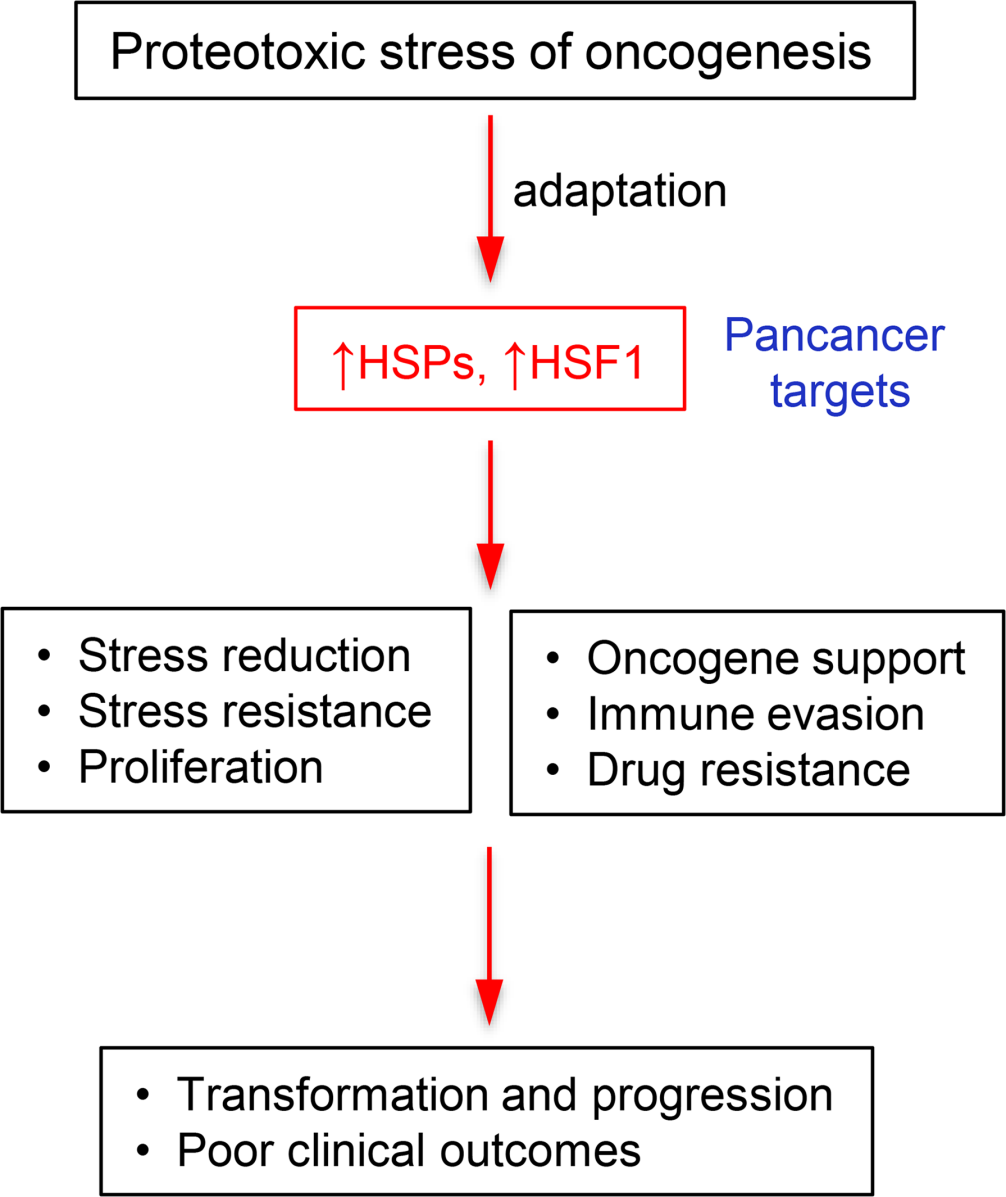
Flow chart depicting the role of heat shock proteins and HSF1 in cancers.

Human cancers almost invariably overexpress main constituents of HSR, including individual HSPs and HSF1. Upregulation and its importance for maintenance of multiple oncogenic pathways identifies HSR as one of pan-cancer targets (**Figure 5**). Preclinical models and more limited clinical studies showed promising results with selected inhibitors of the HSR pathway. A new direction for exploration of antioncogenic effects of HSR inhibition, which has been impossible to detect in the classic tumor xenograft models in immunodeficient mice, is the modulation of immunosuppressive activity by cancers. Combinatory therapy including HSR-targeting drugs offers a potential to enhance the efficacy of clinically used drugs, including immune checkpoint inhibitors. The development of more specific inhibitors with better pharmacokinetic properties is needed for targeting of HSR components, including HSF1, for successful cancer therapy. A strong association with poor prognosis or selective upregulation in malignancy are typically used as the initial screens to identify specific HSR proteins as clinically promising targets for drug development. Another approach for the selection of a drug target can involve identification of HSR components that exhibit a clear pattern of coexpression and/or dependency for a major oncogene that by itself is poorly druggable. Coamplification of HSF1 with a notoriously undruggable MYC oncogene points to HSF1 inhibition as a potential therapeutic strategy for numerous MYC-driven human malignancies. Higher translation activity is necessary to support a rapid growth of transformed cells, but the overabundance of nascent proteins also increases demand for the chaperone/HSR system, which is further exacerbated by aneuploidy-associated excesses in the production of unstable proteins. Thus, it is conceivable that cancers with activating mutations in the protein translation-controlling pathways such as AKT-mTOR can be particularly suitable for HSR-targeting therapies.

## Author Contributions

AC and AZ: conceptualization of the manuscript and editing of the final draft. AC: writing of the first draft. AZ: preparation of the final draft. All authors contributed to the article and approved the submitted version.

## Funding

This work was supported by NIH grants ES031979, ES031002, and ES028072.

## Conflict of Interest

The authors declare that the research was conducted in the absence of any commercial or financial relationships that could be construed as a potential conflict of interest.

## Publisher’s Note

All claims expressed in this article are solely those of the authors and do not necessarily represent those of their affiliated organizations, or those of the publisher, the editors and the reviewers. Any product that may be evaluated in this article, or claim that may be made by its manufacturer, is not guaranteed or endorsed by the publisher.

## Footnotes

1
https://www.picard.ch

## References

[1] Brancolini C Iuliano L. Proteotoxic Stress and Cell Death in Cancer Cells. Cancers (2020) 12(9):2385. doi: 10.3390/cancers12092385 PMC7563887 32842524

[2] Hartl FU Bracher A Hayer-Hartl M. Molecular Chaperones in Protein Folding and Proteostasis. Nature (2011) 475(7356):324–32. doi: 10.1038/nature10317 21776078

[3] Richter K Haslbeck M Buchner J. The Heat Shock Response: Life on the Verge of Death. Mol Cell (2010) 40(2):253–66. doi: 10.1016/j.molcel.2010.10.006 20965420

[4] Akerfelt M Morimoto RI Sistonen L. Heat Shock Factors: Integrators of Cell Stress, Development and Lifespan. Nat Rev Mol Cell Biol (2010) 11(8):545–55. doi: 10.1038/nrm2938 PMC3402356 20628411

[5] Vihervaara A Sergelius C Vasara J Blom MAH Elsing AN Roos-Mattjus P. Transcriptional Response to Stress in the Dynamic Chromatin Environment of Cycling and Mitotic Cells. Proc Natl Acad Sci USA (2013) 110(36):E3388–97. doi: 10.1073/pnas.1305275110 PMC3767495 23959860

[6] Dai C Whitesell L Rogers AB Lindquist S. Heat Shock Factor 1 Is a Powerful Multifaceted Modifier of Carcinogenesis. Cell (2007) 130(6):1005–18. doi: 10.1016/j.cell.2007.07.020 PMC2586609 17889646

[7] Björk JK Åkerfelt M Joutsen J Puustinen MC Cheng F Sistonen L. Heat-Shock Factor 2 is a Suppressor of Prostate Cancer Invasion. Oncogene (2016) 35(14):1770–84. doi: 10.1038/onc.2015.241 PMC4830906 26119944

[8] Ostling P Björk JK Roos-Mattjus P Mezger V Sistonen L. Heat Shock Factor 2 (HSF2) Contributes to Inducible Expression of Hsp Genes Through Interplay With HSF1. J Biol Chem (2007) 282(10):7077–86. doi: 10.1074/jbc.M607556200 17213196

[9] Gomez-Pastor R Burchfiel ET Thiele DJ. Regulation of Heat Shock Transcription Factors and Their Roles in Physiology and Disease. Nat Rev Mol Cell Biol (2018) 19(1):4–19. doi: 10.1038/nrm.2017.73 28852220 PMC5794010

[10] Hanahan D Weinberg RA. The Hallmarks of Cancer. Cell (2000) 100(1):57–70. doi: 10.1016/S0092-8674(00)81683-9 10647931

[11] Hanahan D Weinberg RA. Hallmarks of Cancer: The Next Generation. Cell (2011) 144(5):646–74. doi: 10.1016/j.cell.2011.02.013 21376230

[12] Luo J Solimini NL Elledge SJ. Principles of Cancer Therapy: Oncogene and non-Oncogene Addiction. Cell (2009) 136(5):823–37. doi: 10.1016/j.cell.2009.02.024 PMC2894612 19269363

[13] Weinstein IB. Cancer. Addiction to Oncogenes–the Achilles Heal of Cancer. Science (New York NY) (2002) 297(5578):63–4. doi: 10.1126/science.1073096 12098689

[14] Kampinga HH Hageman J Vos MJ Kubota H Tanguay RM Bruford EA. Guidelines for the Nomenclature of the Human Heat Shock Proteins. Cell Stress Chaperones (2009) 14(1):105–11. doi: 10.1007/s12192-008-0068-7 PMC2673902 18663603

[15] Finka A Goloubinoff P. Proteomic Data From Human Cell Cultures Refine Mechanisms of Chaperone-Mediated Protein Homeostasis. Cell Stress Chaperones (2013) 18(5):591–605. doi: 10.1007/s12192-013-0413-3 23430704 PMC3745260

[16] Saibil H. Chaperone Machines for Protein Folding, Unfolding and Disaggregation. Nat Rev Mol Cell Biol (2013) 14(10):630–42. doi: 10.1038/nrm3658 PMC4340576 24026055

[17] Trepel J Mollapour M Giaccone G Neckers L. Targeting the Dynamic HSP90 Complex in Cancer. Nat Rev Cancer (2010) 10(8):537–49. doi: 10.1038/nrc2887 PMC6778733 20651736

[18] Schopf FH Biebl MM Buchner J. The HSP90 Chaperone Machinery. Nat Rev Mol Cell Biol (2017) 18(6):345–60. doi: 10.1038/nrm.2017.20 28429788

[19] Prodromou C. Mechanisms of Hsp90 Regulation. Biochem J (2016) 473(16):2439–52. doi: 10.1042/BCJ20160005 PMC4980810 27515256

[20] Genest O Wickner S Doyle SM. Hsp90 and Hsp70 Chaperones: Collaborators in Protein Remodeling. J Biol Chem (2019) 294(6):2109–20. doi: 10.1074/jbc.REV118.002806 PMC6369297 30401745

[21] Sahasrabudhe P Rohrberg J Biebl MM Rutz DA Buchner J. The Plasticity of the Hsp90 Co-Chaperone System. Mol Cell (2017) 67(6):947–61.e5. doi: 10.1016/j.molcel.2017.08.004 28890336

[22] Whitesell L Lindquist SL. HSP90 and the Chaperoning of Cancer. Nat Rev Cancer (2005) 5(10):761–72. doi: 10.1038/nrc1716 16175177

[23] Schoof N von Bonin F Trümper L Kube D. HSP90 is Essential for Jak-STAT Signaling in Classical Hodgkin Lymphoma Cells. Cell Communication Signaling (2009) 7(1):17. doi: 10.1186/1478-811X-7-17 19607667 PMC2714310

[24] Moulick K Ahn JH Zong H Rodina A Cerchietti L Gomes DaGama EM. Affinity-Based Proteomics Reveal Cancer-Specific Networks Coordinated by Hsp90. Nat Chem Biol (2011) 7(11):818–26. doi: 10.1038/nchembio.670 PMC3265389 21946277

[25] Tang Z Li C Kang B Gao G Li C Zhang Z. GEPIA: A Web Server for Cancer and Normal Gene Expression Profiling and Interactive Analyses. Nucleic Acids Res (2017) 45(W1):W98–102. doi: 10.1093/nar/gkx247 28407145 PMC5570223

[26] Sanchez J Carter TR Cohen MS Blagg BSJ. Old and New Approaches to Target the Hsp90 Chaperone. Curr Cancer Drug Targets (2020) 20(4):253–70. doi: 10.2174/1568009619666191202101330 PMC7502213 31793427

[27] Samuni Y Ishii H Hyodo F Samuni U Krishna MC Goldstein S. Reactive Oxygen Species Mediate Hepatotoxicity Induced by the Hsp90 Inhibitor Geldanamycin and its Analogs. Free Radical Biol Med (2010) 48(11):1559–63. doi: 10.1016/j.freeradbiomed.2010.03.001 PMC2862863 20211249

[28] Shevtsov M Multhoff G Mikhaylova E Shibata A Guzhova I Margulis B. Combination of Anti-Cancer Drugs With Molecular Chaperone Inhibitors. Int J Mol Sci (2019) 20(21):5284. doi: 10.3390/ijms20215284 PMC6862641 31652993

[29] Modi S Sugarman S Stopeck A Linden H Ma W Kersey K. Phase II Trial of the Hsp90 Inhibitor Tanespimycin (Tan) + Trastuzumab (T) in Patients (Pts) With HER2-Positive Metastatic Breast Cancer (MBC). J Clin Oncol (2008) 26(15_suppl):1027. doi: 10.1200/jco.2008.26.15_suppl.1027 18309938

[30] Ronnen EA Kondagunta GV Ishill N Sweeney SM DeLuca JK Schwartz L. A Phase II Trial of 17-(Allylamino)-17-Demethoxygeldanamycin in Patients With Papillary and Clear Cell Renal Cell Carcinoma. Invest New Drugs (2006) 24(6):543–6. doi: 10.1007/s10637-006-9208-z 16832603

[31] Heath EI Hillman DW Vaishampayan U Sheng S Sarkar F Harper F. A Phase II Trial of 17-Allylamino-17-Demethoxygeldanamycin in Patients With Hormone-Refractory Metastatic Prostate Cancer. Clin Cancer Res (2008) 14(23):7940–6. doi: 10.1158/1078-0432.CCR-08-0221 PMC3085545 19047126

[32] Talaei S Mellatyar H Asadi A Akbarzadeh A Sheervalilou R Zarghami N. Spotlight on 17-AAG as an Hsp90 Inhibitor for Molecular Targeted Cancer Treatment. Chem Biol Drug Design (2019) 93(5):760–86. doi: 10.1111/cbdd.13486 30697932

[33] Cavenagh J Oakervee H Baetiong-Caguioa P Davies F Gharibo M Rabin N. A Phase I/II Study of KW-2478, an Hsp90 Inhibitor, in Combination With Bortezomib in Patients With Relapsed/Refractory Multiple Myeloma. Br J Cancer (2017) 117(9):1295–302. doi: 10.1038/bjc.2017.302 PMC5672925 28873084

[34] Banerji U Walton M Raynaud F Grimshaw R Kelland L Valenti M. Pharmacokinetic-Pharmacodynamic Relationships for the Heat Shock Protein 90 Molecular Chaperone Inhibitor 17-Allylamino, 17-Demethoxygeldanamycin in Human Ovarian Cancer Xenograft Models. Clin Cancer Res (2005) 11(19):7023–32. doi: 10.1158/1078-0432.CCR-05-0518 16203796

[35] Brocchieri L Conway de Macario E Macario AJL. Hsp70 Genes in the Human Genome: Conservation and Differentiation Patterns Predict a Wide Array of Overlapping and Specialized Functions. BMC Evol Biol (2008) 8(1):19. doi: 10.1186/1471-2148-8-19 18215318 PMC2266713

[36] Nitika Porter CM Truman AW Truttmann MC. Post-Translational Modifications of Hsp70 Family Proteins: Expanding the Chaperone Code. J Biol Chem (2020) 295(31):10689–708. doi: 10.1074/jbc.REV120.011666 PMC7397107 32518165

[37] Radons J. The Human HSP70 Family of Chaperones: Where do We Stand? Cell Stress Chaperones (2016) 21(3):379–404. doi: 10.1007/s12192-016-0676-6 26865365 PMC4837186

[38] Zorzi E Bonvini P. Inducible Hsp70 in the Regulation of Cancer Cell Survival: Analysis of Chaperone Induction, Expression and Activity. Cancers (2011) 3(4):3921–56. doi: 10.3390/cancers3043921 PMC3763403 24213118

[39] Kampinga HH Craig EA. The HSP70 Chaperone Machinery: J Proteins as Drivers of Functional Specificity. Nat Rev Mol Cell Biol (2010) 11(8):579–92. doi: 10.1038/nrm2941 PMC3003299 20651708

[40] Wandinger SK Richter K Buchner J. The Hsp90 Chaperone Machinery. J Biol Chem (2008) 283(27):18473–7. doi: 10.1074/jbc.R800007200 18442971

[41] Finka A Sharma SK Goloubinoff P. Multi-Layered Molecular Mechanisms of Polypeptide Holding, Unfolding and Disaggregation by HSP70/HSP110 Chaperones. Front Mol Biosci (2015) 2:29. doi: 10.3389/fmolb.2015.00029 26097841 PMC4456865

[42] Bracher A Verghese J. GrpE, Hsp110/Grp170, HspBP1/Sil1 and BAG Domain Proteins: Nucleotide Exchange Factors for Hsp70 Molecular Chaperones. Sub Cellular Biochem (2015) 78:1–33. doi: 10.1007/978-3-319-11731-7_1 25487014

[43] Bracher A Verghese J. The Nucleotide Exchange Factors of Hsp70 Molecular Chaperones. Front Mol Biosci (2015) 2:10. doi: 10.3389/fmolb.2015.00010 26913285 PMC4753570

[44] Shen C Kaelin WG Jr. The VHL/HIF Axis in Clear Cell Renal Carcinoma. Semin Cancer Biol (2013) 23(1):18–25. doi: 10.1016/j.semcancer.2012.06.001 22705278 PMC3663044

[45] Jagadish N Parashar D Gupta N Agarwal S Suri V Kumar R. Heat Shock Protein 70-2 (HSP70-2) is a Novel Therapeutic Target for Colorectal Cancer and is Associated With Tumor Growth. BMC Cancer (2016) 16:561. doi: 10.1186/s12885-016-2592-7 27473057 PMC4966739

[46] Garg M Kanojia D Seth A Kumar R Gupta A Surolia A. Heat-Shock Protein 70-2 (HSP70-2) Expression in Bladder Urothelial Carcinoma is Associated With Tumour Progression and Promotes Migration and Invasion. Eur J Cancer (Oxford England: 1990) (2010) 46(1):207–15. doi: 10.1016/j.ejca.2009.10.020 19914824

[47] Singh S Suri A. Targeting the Testis-Specific Heat-Shock Protein 70-2 (HSP70-2) Reduces Cellular Growth, Migration, and Invasion in Renal Cell Carcinoma Cells. Tumour Biol (2014) 35(12):12695–706. doi: 10.1007/s13277-014-2594-5 25213699

[48] Aghdassi A Phillips P Dudeja V Dhaulakhandi D Sharif R Dawra R. Heat Shock Protein 70 Increases Tumorigenicity and Inhibits Apoptosis in Pancreatic Adenocarcinoma. Cancer Res (2007) 67(2):616–25. doi: 10.1158/0008-5472.CAN-06-1567 17234771

[49] Rohde M Daugaard M Jensen MH Helin K Nylandsted J Jäättelä M. Members of the Heat-Shock Protein 70 Family Promote Cancer Cell Growth by Distinct Mechanisms. Genes Dev (2005) 19(5):570–82. doi: 10.1101/gad.305405 PMC551577 15741319

[50] Wang B Lan T Xiao H Chen Z-H Wei C Chen L-F. The Expression Profiles and Prognostic Values of HSP70s in Hepatocellular Carcinoma. Cancer Cell Int (2021) 21(1):286. doi: 10.1186/s12935-021-01987-9 34059060 PMC8165812

[51] Stankiewicz AR Lachapelle G Foo CPZ Radicioni SM Mosser DD. Hsp70 Inhibits Heat-Induced Apoptosis Upstream of Mitochondria by Preventing Bax Translocation. J Biol Chem (2005) 280(46):38729–39. doi: 10.1074/jbc.M509497200 16172114

[52] Li H Liu L Xing D Chen WR. Inhibition of the JNK/Bim Pathway by Hsp70 Prevents Bax Activation in UV-Induced Apoptosis. FEBS Lett (2010) 584(22):4672–8. doi: 10.1016/j.febslet.2010.10.050 PMC3397246 21034742

[53] Nylandsted J Gyrd-Hansen M Danielewicz A Fehrenbacher N Lademann U Høyer-Hansen M. Heat Shock Protein 70 Promotes Cell Survival by Inhibiting Lysosomal Membrane Permeabilization. J Exp Med (2004) 200(4):425–35. doi: 10.1084/jem.20040531 PMC2211935 15314073

[54] Guo F Sigua C Bali P George P Fiskus W Scuto A. Mechanistic Role of Heat Shock Protein 70 in Bcr-Abl-Mediated Resistance to Apoptosis in Human Acute Leukemia Cells. Blood (2005) 105(3):1246–55. doi: 10.1182/blood-2004-05-2041 15388581

[55] Zhuang H Jiang W Zhang X Qiu F Gan Z Cheng W. Suppression of HSP70 Expression Sensitizes NSCLC Cell Lines to TRAIL-Induced Apoptosis by Upregulating DR4 and DR5 and Downregulating C-FLIP-L Expressions. J Mol Med (Berlin Germany) (2013) 91(2):219–35. doi: 10.1007/s00109-012-0947-3 22948392

[56] Park H-S Cho S-G Kim CK Hwang HS Noh KT Kim M-S. Heat Shock Protein Hsp72 Is a Negative Regulator of Apoptosis Signal-Regulating Kinase 1. Mol Cell Biol (2002) 22(22):7721–30. doi: 10.1128/MCB.22.22.7721-7730.2002 PMC134722 12391142

[57] Gabai VL Yaglom JA Waldman T Sherman MY. Heat Shock Protein Hsp72 Controls Oncogene-Induced Senescence Pathways in Cancer Cells. Mol Cell Biol (2009) 29(2):559–69. doi: 10.1128/MCB.01041-08 PMC2612502 19001088

[58] Meng L Hunt C Yaglom JA Gabai VL Sherman MY. Heat Shock Protein Hsp72 Plays an Essential Role in Her2-Induced Mammary Tumorigenesis. Oncogene (2011) 30(25):2836–45. doi: 10.1038/onc.2011.5 PMC3433756 21297664

[59] Albakova Z Armeev GA Kanevskiy LM Kovalenko EI Sapozhnikov AM. HSP70 Multi-Functionality in Cancer. Cells (2020) 9(3):587. doi: 10.3390/cells9030587 PMC7140411 32121660

[60] Moradi-Marjaneh R Paseban M Marjaneh MM. Hsp70 Inhibitors: Implications for the Treatment of Colorectal Cancer. IUBMB Life (2019) 71:1834–45. doi: 10.1002/iub.2157 31441584

[61] Goloudina AR Demidov ON Garrido C. Inhibition of HSP70: A Challenging Anti-Cancer Strategy. Cancer Lett [Internet] (2012) 325(2):117–24. doi: 10.1016/j.canlet.2012.06.003 22750096

[62] Li X Srinivasan SR Connarn J Ahmad A Young ZT Kabza AM. Analogs of the Allosteric Heat Shock Protein 70 (Hsp70) Inhibitor, MKT-077, as Anti-Cancer Agents. ACS Medicinal Chem Lett (2013) 4(11):1042–7. doi: 10.1021/ml400204n PMC3845967 24312699

[63] Davis AL Cabello CM Qiao S Azimian S Wondrak GT. Phenotypic Identification of the Redox Dye Methylene Blue as an Antagonist of Heat Shock Response Gene Expression in Metastatic Melanoma Cells. Int J Mol Sci (2013) 14(2):4185–202. doi: 10.3390/ijms14024185 PMC3588094 23429201

[64] Qiu X-B Shao Y-M Miao S Wang L. The Diversity of the DnaJ/Hsp40 Family, the Crucial Partners for Hsp70 Chaperones. Cell Mol Life Sci (2006) 63(22):2560–70. doi: 10.1007/s00018-006-6192-6 PMC11136209 16952052

[65] Fan C-Y Lee S Ren H-Y Cyr DM. Exchangeable Chaperone Modules Contribute to Specification of Type I and Type II Hsp40 Cellular Function. Mol Biol Cell (2004) 15(2):761–73. doi: 10.1091/mbc.e03-03-0146 PMC329391 14657253

[66] Cheetham ME Caplan AJ. Structure, Function and Evolution of DnaJ: Conservation and Adaptation of Chaperone Function. Cell Stress Chaperones (1998) 3(1):28–36. doi: 10.1379/1466-1268(1998)003<0028:SFAEOD>2.3.CO;2 9585179 PMC312945

[67] Tsai M-F Wang C-C Chen JJ. Tumour Suppressor HLJ1: A Potential Diagnostic, Preventive and Therapeutic Target in Non-Small Cell Lung Cancer. World J Clin Oncol (2014) 5(5):865–73. doi: 10.5306/wjco.v5.i5.865 PMC4259948 25493224

[68] Lin S-Y Hsueh C-M Yu S-L Su C-C Shum W-Y Yeh K-C. HLJ1 Is a Novel Caspase-3 Substrate and its Expression Enhances UV-Induced Apoptosis in non-Small Cell Lung Carcinoma. Nucleic Acids Res (2010) 38(18):6148–58. doi: 10.1093/nar/gkq412 PMC2952861 20494979

[69] Chen H-W Lee J-Y Huang J-Y Wang C-C Chen W-J Su S-F. Curcumin Inhibits Lung Cancer Cell Invasion and Metastasis Through the Tumor Suppressor HLJ1. Cancer Res (2008) 68(18):7428–38. doi: 10.1158/0008-5472.CAN-07-6734 18794131

[70] Tsai M-F Wang C-C Chang G-C Chen C-Y Chen H-Y Cheng C-L. A New Tumor Suppressor DnaJ-Like Heat Shock Protein, HLJ1, and Survival of Patients With Non–Small-Cell Lung Carcinoma. JNCI: J Natl Cancer Institute (2006) 98(12):825–38. doi: 10.1093/jnci/djj229 16788156

[71] Wang C-C Tsai M-F Hong T-M Chang G-C Chen C-Y Yang W-M. The Transcriptional Factor YY1 Upregulates the Novel Invasion Suppressor HLJ1 Expression and Inhibits Cancer Cell Invasion. Oncogene (2005) 24(25):4081–93. doi: 10.1038/sj.onc.1208573 15782117

[72] Acun T Doberstein N Habermann JK Gemoll T Thorns C Oztas E. HLJ1 (DNAJB4) Gene Is a Novel Biomarker Candidate in Breast Cancer. Omics (2017) 21(5):257–65. doi: 10.1089/omi.2017.0016 PMC5586162 28481734

[73] Chen C-H Chang W-H Su K-Y Ku W-H Chang G-C Hong Q-S. HLJ1 is an Endogenous Src Inhibitor Suppressing Cancer Progression Through Dual Mechanisms. Oncogene (2016) 35(43):5674–85. doi: 10.1038/onc.2016.106 27065329

[74] Schwanhäusser B Busse D Li N Dittmar G Schuchhardt J Wolf J. Global Quantification of Mammalian Gene Expression Control. Nature (2011) 473(7347):337–42. doi: 10.1038/nature10098 21593866

[75] Kosti I Jain N Aran D Butte AJ Sirota M. Cross-Tissue Analysis of Gene and Protein Expression in Normal and Cancer Tissues. Sci Rep (2016) 6:24799. doi: 10.1038/srep24799 27142790 PMC4855174

[76] Okamoto T Ishida R Yamamoto H Tanabe-Ishida M Haga A Takahashi H. Functional Structure and Physiological Functions of Mammalian Wild-Type HSP60. Arch Biochem Biophys (2015) 586:10–9. doi: 10.1016/j.abb.2015.09.022 26427351

[77] Pace A Barone G Lauria A Martorana A Piccionello AP Pierro P. Hsp60, a Novel Target for Antitumor Therapy: Structure-Function Features and Prospective Drugs Design. Curr Pharm Design (2013) 19(15):2757–64. doi: 10.2174/1381612811319150011 23092316

[78] Merendino AM Bucchieri F Campanella C Marcianò V Ribbene A David S. Hsp60 is Actively Secreted by Human Tumor Cells. PLoS One (2010) 5(2):e9247. doi: 10.1371/journal.pone.0009247 20169074 PMC2821922

[79] Cappello F Conway de Macario E Marasà L Zummo G Macario AJL. Hsp60 Expression, New Locations, Functions and Perspectives for Cancer Diagnosis and Therapy. Cancer Biol Ther (2008) 7(6):801–9. doi: 10.4161/cbt.7.6.6281 18497565

[80] Quintana FJ Cohen IR. The HSP60 Immune System Network. Trends Immunol (2011) 32(2):89–95. doi: 10.1016/j.it.2010.11.001 21145789

[81] Cohen-Sfady M Nussbaum G Pevsner-Fischer M Mor F Carmi P Zanin-Zhorov A. Heat Shock Protein 60 Activates B Cells *via* the TLR4-MyD88 Pathway. J Immunol (Baltimore Md: 1950) (2005) 175(6):3594–602. doi: 10.4049/jimmunol.175.6.3594 16148103

[82] Li S Ma W Fei T Lou Q Zhang Y Cui X. Upregulation of Heat Shock Factor 1 Transcription Activity is Associated With Hepatocellular Carcinoma Progression. Mol Med Rep (2014) 10(5):2313–21. doi: 10.3892/mmr.2014.2547 PMC4214332 25199534

[83] Castilla C Congregado B Conde JM Medina R Torrubia FJ Japón MA. Immunohistochemical Expression of Hsp60 Correlates With Tumor Progression and Hormone Resistance in Prostate Cancer. Urology (2010) 76(4):1017. doi: 10.1016/j.urology.2010.05.045 20708221

[84] Li X Xu Q Fu X Luo W. Heat Shock Protein 60 Overexpression is Associated With the Progression and Prognosis in Gastric Cancer. PLoS One (2014) 9(9):e107507. doi: 10.1371/journal.pone.0107507 25207654 PMC4160299

[85] Lebret T Watson RWG Molinié V O’Neill A Gabriel C Fitzpatrick JM. Heat Shock Proteins HSP27, HSP60, HSP70, and HSP90. Cancer (2003) 98(5):970–7. doi: 10.1002/cncr.11594 12942564

[86] Guo J Li X Zhang W Chen Y Zhu S Chen L. HSP60-Regulated Mitochondrial Proteostasis and Protein Translation Promote Tumor Growth of Ovarian Cancer. Sci Rep (2019) 9(1):12628. doi: 10.1038/s41598-019-48992-7 31477750 PMC6718431

[87] Zhou C Sun H Zheng C Gao J Fu Q Hu N. Oncogenic HSP60 Regulates Mitochondrial Oxidative Phosphorylation to Support Erk1/2 Activation During Pancreatic Cancer Cell Growth. Cell Death Dis (2018) 9(2):161. doi: 10.1038/s41419-017-0196-z 29415987 PMC5833694

[88] Kim W Ryu J Kim J-E. CCAR2/DBC1 and Hsp60 Positively Regulate Expression of Survivin in Neuroblastoma Cells. Int J Mol Sci (2019) 20(1):131. doi: 10.3390/ijms20010131 PMC6337645 30609639

[89] Hua Shu J Tadashi Y Takashi O Keita K Rie Y Yi Xuan L. Expression of Heat Shock Protein 60 in Normal and Neoplastic Human Lymphoid Tissues. J Clin Exp Hematopathol (2002) 42(1):25–32. doi: 10.3960/jslrt.42.25

[90] Hamelin C Cornut E Poirier F Pons S Beaulieu C Charrier J-P. Identification and Verification of Heat Shock Protein 60 as a Potential Serum Marker for Colorectal Cancer. FEBS J (2011) 278(24):4845–59. doi: 10.1111/j.1742-4658.2011.08385.x PMC3265716 21973086

[91] Tsai Y-P Teng S-C Wu K-J. Direct Regulation of HSP60 Expression by C-MYC Induces Transformation. FEBS Lett (2008) 582(29):4083–8. doi: 10.1016/j.febslet.2008.11.004 19022255

[92] Chun JN Choi B Lee KW Lee DJ Kang DH Lee JY. Cytosolic Hsp60 is Involved in the NF-kappaB-Dependent Survival of Cancer Cells *via* IKK Regulation. PLoS One (2010) 5(3):e9422. doi: 10.1371/journal.pone.0009422 20351780 PMC2843631

[93] Kumar S O’Malley J Chaudhary AK Inigo JR Yadav N Kumar R. Hsp60 and IL-8 Axis Promotes Apoptosis Resistance in Cancer. Br J Cancer (2019) 121(11):934–43. doi: 10.1038/s41416-019-0617-0 PMC6889399 31673102

[94] Ghosh JC Dohi T Kang BH Altieri DC. Hsp60 Regulation of Tumor Cell Apoptosis. J Biol Chem (2008) 283(8):5188–94. doi: 10.1074/jbc.M705904200 18086682

[95] Marino Gammazza A Campanella C Barone R Caruso Bavisotto C Gorska M Wozniak M. Doxorubicin Anti-Tumor Mechanisms Include Hsp60 Post-Translational Modifications Leading to the Hsp60/p53 Complex Dissociation and Instauration of Replicative Senescence. Cancer Lett (2017) 385:75–86. doi: 10.1016/j.canlet.2016.10.045 27836734

[96] Chandra D Choy G Tang DG. Cytosolic Accumulation of HSP60 During Apoptosis With or Without Apparent Mitochondrial Release: Evidence That its Pro-Apoptotic or Pro-Survival Functions Involve Differential Interactions With Caspase-3. J Biol Chem (2007) 282(43):31289–301. doi: 10.1074/jbc.M702777200 17823127

[97] Tang H Li J Liu X Wang G Luo M Deng H. Down-Regulation of HSP60 Suppresses the Proliferation of Glioblastoma Cells *via* the ROS/AMPK/mTOR Pathway. Sci Rep (2016) 6:28388. doi: 10.1038/srep28388 27325206 PMC4914999

[98] Tsai Y-P Yang M-H Huang C-H Chang S-Y Chen P-M Liu C-J. Interaction Between HSP60 and Beta-Catenin Promotes Metastasis. Carcinogenesis (2009) 30(6):1049–57. doi: 10.1093/carcin/bgp087 19369584

[99] Barazi HO Zhou L Templeton NS Krutzsch HC Roberts DD. Identification of Heat Shock Protein 60 as a Molecular Mediator of Alpha 3 Beta 1 Integrin Activation. Cancer Res (2002) 62(5):1541–8. 11888933

[100] Tang H Chen Y Liu X Wang S Lv Y Wu D. Downregulation of HSP60 Disrupts Mitochondrial Proteostasis to Promote Tumorigenesis and Progression in Clear Cell Renal Cell Carcinoma. Oncotarget (2016) 7(25):38822–34. doi: 10.18632/oncotarget.9615 PMC5122432 27246978

[101] Rappa F Pitruzzella A Marino Gammazza A Barone R Mocciaro E Tomasello G. Quantitative Patterns of Hsps in Tubular Adenoma Compared With Normal and Tumor Tissues Reveal the Value of Hsp10 and Hsp60 in Early Diagnosis of Large Bowel Cancer. Cell Stress Chaperones (2016) 21(5):927–33. doi: 10.1007/s12192-016-0721-5 PMC5003810 27491302

[102] Vocka M Langer D Fryba V Petrtyl J Hanus T Kalousova M. Novel Serum Markers HSP60, CHI3L1, and IGFBP-2 in Metastatic Colorectal Cancer. Oncol Lett (2019) 18(6):6284–92. doi: 10.3892/ol.2019.10925 PMC6864964 31788106

[103] Zhang J Zhou X Chang H Huang X Guo X Du X. Hsp60 Exerts a Tumor Suppressor Function by Inducing Cell Differentiation and Inhibiting Invasion in Hepatocellular Carcinoma. Oncotarget (2016) 7(42):68976–89. doi: 10.18632/oncotarget.12185 PMC5356605 27677587

[104] Nakamura H Minegishi H. HSP60 as a Drug Target. Curr Pharm Design (2013) 19(3):441–51. doi: 10.2174/138161213804143626 22920899

[105] Xu X Wang W Shao W Yin W Chen H Qiu Y. Heat Shock Protein-60 Expression was Significantly Correlated With the Prognosis of Lung Adenocarcinoma. J Surg Oncol (2011) 104(6):598–603. doi: 10.1002/jso.21992 21671464

[106] Ağababaoğlu İ Önen A Demir AB Aktaş S Altun Z Ersöz H. Chaperonin (HSP60) and Annexin-2 are Candidate Biomarkers for non-Small Cell Lung Carcinoma. Medicine (2017) 96(6):e5903. doi: 10.1097/MD.0000000000005903 28178129 PMC5312986

[107] Desmetz C Bibeau F Boissière F Bellet V Rouanet P Maudelonde T. Proteomics-Based Identification of HSP60 as a Tumor-Associated Antigen in Early Stage Breast Cancer and Ductal Carcinoma in Situ. J Proteome Res (2008) 7(9):3830–7. doi: 10.1021/pr800130d 18683965

[108] Trieb K Gerth R Windhager R Grohs JG Holzer G Berger P. Serum Antibodies Against the Heat Shock Protein 60 are Elevated in Patients With Osteosarcoma. Immunobiology (2000) 201(3–4):368–76. doi: 10.1016/S0171-2985(00)80091-1 10776793

[109] Itoh H Komatsuda A Wakui H Miura AB Tashima Y. Mammalian HSP60 is a Major Target for an Immunosuppressant Mizoribine. J Biol Chem (1999) 274(49):35147–51. doi: 10.1074/jbc.274.49.35147 10574997

[110] Tanabe M Ishida R Izuhara F Komatsuda A Wakui H Sawada K. The ATPase Activity of Molecular Chaperone HSP60 is Inhibited by Immunosuppressant Mizoribine. Am J Mol Biol (2012) 2:93–102. doi: 10.4236/ajmb.2012.22010

[111] Nagumo Y Kakeya H Shoji M Hayashi Y Dohmae N Osada H. Epolactaene Binds Human Hsp60 Cys442 Resulting in the Inhibition of Chaperone Activity. Biochem J (2005) 387(Pt 3):835–40. doi: 10.1042/BJ20041355 PMC1135015 15603555

[112] Izgi K Iskender B Jauch J Sezen S Cakir M Charpentier M. Myrtucommulone-A Induces Both Extrinsic and Intrinsic Apoptotic Pathways in Cancer Cells. J Biochem Mol Toxicol (2015) 29(9):432–9. doi: 10.1002/jbt.21716 26032814

[113] Wiechmann K Müller H König S Wielsch N Svatoš A Jauch J. Mitochondrial Chaperonin HSP60 Is the Apoptosis-Related Target for Myrtucommulone. Cell Chem Biol (2017) 24(5):614–23. doi: 10.1016/j.chembiol.2017.04.008 28457707

[114] Ban HS Shimizu K Minegishi H Nakamura H. Identification of HSP60 as a Primary Target of O-Carboranylphenoxyacetanilide, an HIF-1alpha Inhibitor. J Am Chem Society (2010) 132(34):11870–1. doi: 10.1021/ja104739t 20695501

[115] Meng Q Li BX Xiao X. Toward Developing Chemical Modulators of Hsp60 as Potential Therapeutics. Front Mol Biosci (2018) 5:35. doi: 10.3389/fmolb.2018.00035 29732373 PMC5920047

[116] Zoubeidi A Gleave M. Small Heat Shock Proteins in Cancer Therapy and Prognosis. Int J Biochem Cell Biol (2012) 44(10):1646–56. doi: 10.1016/j.biocel.2012.04.010 22571949

[117] Carra S Alberti S Arrigo PA Benesch JL Benjamin IJ Boelens W. The Growing World of Small Heat Shock Proteins: From Structure to Functions. Cell Stress Chaperones (2017) 22(4):601–11. doi: 10.1007/s12192-017-0787-8 PMC5465036 28364346

[118] Haslbeck M Vierling E. A First Line of Stress Defense: Small Heat Shock Proteins and Their Function in Protein Homeostasis. J Mol Biol (2015) 427(7):1537–48. doi: 10.1016/j.jmb.2015.02.002 PMC4360138 25681016

[119] Kostenko S Moens U. Heat Shock Protein 27 Phosphorylation: Kinases, Phosphatases, Functions and Pathology. Cell Mol Life Sci (2009) 66(20):3289–307. doi: 10.1007/s00018-009-0086-3 PMC11115724 19593530

[120] Yu Z Zhi J Peng X Zhong X Xu A. Clinical Significance of HSP27 Expression in Colorectal Cancer. Mol Med Rep (2010) 3(6):953–8. doi: 10.3892/mmr.2010.372 21472339

[121] Spigel DR Shipley DL Waterhouse DM Jones SF Ward PJ Shih KC. A Randomized, Double-Blinded, Phase II Trial of Carboplatin and Pemetrexed With or Without Apatorsen (OGX-427) in Patients With Previously Untreated Stage IV Non-Squamous-Non-Small-Cell Lung Cancer: The SPRUCE Trial. Oncol (2019) 24(12):e1409–16. doi: 10.1634/theoncologist.2018-0518 PMC6975937 31420467

[122] Ko AH Murphy PB Peyton JD Shipley DL Al-Hazzouri A Rodriguez FA. A Randomized, Double-Blinded, Phase II Trial of Gemcitabine and Nab-Paclitaxel Plus Apatorsen or Placebo in Patients With Metastatic Pancreatic Cancer: The RAINIER Trial. Oncol (2017) 22(12):1427–e129. doi: 10.1634/theoncologist.2017-0066 PMC5728028 28935773

[123] Bellmunt J Eigl BJ Senkus E Loriot Y Twardowski P Castellano D. Borealis-1: A Randomized, First-Line, Placebo-Controlled, Phase II Study Evaluating Apatorsen and Chemotherapy for Patients With Advanced Urothelial Cancer. Ann Oncol (2017) 28(10):2481–8. doi: 10.1093/annonc/mdx400 28961845

[124] Rosenberg JE Hahn NM Regan MM Werner L Alva A George S. Apatorsen Plus Docetaxel Versus Docetaxel Alone in Platinum-Resistant Metastatic Urothelial Carcinoma (Borealis-2). Br J Cancer (2018) 118(11):1434–41. doi: 10.1038/s41416-018-0087-9 PMC5988804 29765151

[125] Pirkkala L Nykanen P Sistonen L. Roles of the Heat Shock Transcription Factors in Regulation of the Heat Shock Response and Beyond. FASEB J (2001) 15(7):1118–31. doi: 10.1096/fj00-0294rev 11344080

[126] Li J Labbadia J Morimoto RI. Rethinking HSF1 in Stress, Development, and Organismal Health. Trends Cell Biol (2017) 27(12):895–905. doi: 10.1016/j.tcb.2017.08.002 28890254 PMC5696061

[127] Guo Y Guettouche T Fenna M Boellmann F Pratt WB Toft DO. Evidence for a Mechanism of Repression of Heat Shock Factor 1 Transcriptional Activity by a Multichaperone Complex. J Biol Chem (2001) 276(49):45791–9. doi: 10.1074/jbc.M105931200 11583998

[128] Anckar J Sistonen L. Regulation of HSF1 Function in the Heat Stress Response: Implications in Aging and Disease. Annu Rev Biochem (2011) 80(1):1089–115. doi: 10.1146/annurev-biochem-060809-095203 21417720

[129] Hentze N Le Breton L Wiesner J Kempf G Mayer MP. Molecular Mechanism of Thermosensory Function of Human Heat Shock Transcription Factor Hsf1. eLife (2016) 5:e11576. doi: 10.7554/eLife.11576.017 26785146 PMC4775227

[130] Ahn SG Liu PC Klyachko K Morimoto RI Thiele DJ. The Loop Domain of Heat Shock Transcription Factor 1 Dictates DNA-Binding Specificity and Responses to Heat Stress. Genes Dev (2001) 15(16):2134–45. doi: 10.1101/gad.894801 PMC312766 11511544

[131] Shamovsky I Ivannikov M Kandel ES Gershon D Nudler E. RNA-Mediated Response to Heat Shock in Mammalian Cells. Nature (2006) 440(7083):556–60. doi: 10.1038/nature04518 16554823

[132] Jaeger AM Makley LN Gestwicki JE Thiele DJ. Genomic Heat Shock Element Sequences Drive Cooperative Human Heat Shock Factor 1 DNA Binding and Selectivity*. J Biol Chem (2014) 289(44):30459–69. doi: 10.1074/jbc.M114.591578 PMC4215228 25204655

[133] Mahat DB Salamanca HH Duarte FM Danko CG Lis JT. Mammalian Heat Shock Response and Mechanisms Underlying Its Genome-Wide Transcriptional Regulation. Mol Cell (2016) 62(1):63–78. doi: 10.1016/j.molcel.2016.02.025 27052732 PMC4826300

[134] Elsing AN Aspelin C Bjork JK Bergman HA Himanen SV Kallio MJ. Expression of HSF2 Decreases in Mitosis to Enable Stress-Inducible Transcription and Cell Survival. J Cell Biol (2014) 206(6):735–49. doi: 10.1083/jcb.201402002 PMC4164949 25202032

[135] Neueder A Gipson TA Batterton S Lazell HJ Farshim PP Paganetti P. HSF1-Dependent and -Independent Regulation of the Mammalian *In Vivo* Heat Shock Response and its Impairment in Huntington’s Disease Mouse Models. Sci Rep (2017) 7(1):12556. doi: 10.1038/s41598-017-12897-0 28970536 PMC5624871

[136] Duarte FM Fuda NJ Mahat DB Core LJ Guertin MJ Lis JT. Transcription Factors GAF and HSF Act at Distinct Regulatory Steps to Modulate Stress-Induced Gene Activation. Genes Dev (2016) 30(15):1731–46. doi: 10.1101/gad.284430.116 PMC5002978 27492368

[137] Neueder A Achilli F Moussaoui S Bates GP. Novel Isoforms of Heat Shock Transcription Factor 1, HSF1γα and HSF1γβ, Regulate Chaperone Protein Gene Transcription. J Biol Chem (2014) 289(29):19894–906. doi: 10.1074/jbc.M114.570739 PMC4106310 24855652

[138] Su K-H Cao J Tang Z Dai S He Y Sampson SB. HSF1 Critically Attunes Proteotoxic Stress Sensing by Mtorc1 to Combat Stress and Promote Growth. Nat Cell Biol (2016) 18(5):527–39. doi: 10.1038/ncb3335 PMC5341796 27043084

[139] Kmiecik SW Le Breton L Mayer MP. Feedback Regulation of Heat Shock Factor 1 (Hsf1) Activity by Hsp70-Mediated Trimer Unzipping and Dissociation From DNA. EMBO J (2020) 39(14):e104096. doi: 10.15252/embj.2019104096 32490574 PMC7360973

[140] Ding XZ Tsokos GC Kiang JG. Overexpression of HSP-70 Inhibits the Phosphorylation of HSF1 by Activating Protein Phosphatase and Inhibiting Protein Kinase C Activity. FASEB J (1998) 12(6):451–9. doi: 10.1096/fasebj.12.6.451 9535217

[141] Dayalan Naidu S Sutherland C Zhang Y Risco A de la Vega L Caunt CJ. Heat Shock Factor 1 Is a Substrate for P38 Mitogen-Activated Protein Kinases. Mol Cell Biol (2016) 36(18):2403–17. doi: 10.1128/MCB.00292-16 PMC5007788 27354066

[142] Xu Y-M Huang D-Y Chiu J-F Lau ATY. Post-Translational Modification of Human Heat Shock Factors and Their Functions: A Recent Update by Proteomic Approach. J Proteome Res (2012) 11(5):2625–34. doi: 10.1021/pr201151a 22494029

[143] Soncin F Zhang X Chu B Wang X Asea A Ann Stevenson M. Transcriptional Activity and DNA Binding of Heat Shock Factor-1 Involve Phosphorylation on Threonine 142 by CK2. Biochem Biophys Res Commun (2003) 303(2):700–6. doi: 10.1016/S0006-291X(03)00398-X 12659875

[144] Guettouche T Boellmann F Lane WS Voellmy R. Analysis of Phosphorylation of Human Heat Shock Factor 1 in Cells Experiencing a Stress. BMC Biochem (2005) 6:4. doi: 10.1186/1471-2091-6-4 15760475 PMC1079796

[145] Murshid A Chou S-D Prince T Zhang Y Bharti A Calderwood SK. Protein Kinase A Binds and Activates Heat Shock Factor 1. PLoS One (2010) 5(11):1–13. doi: 10.1371/annotation/5879464d-8556-4c3e-b11c-a96cbbff44a6 PMC2976705 21085490

[146] Lu WC Omari R Ray H Wang J Williams I Jacobs C. AKT1 Mediates Multiple Phosphorylation Events That Functionally Promote HSF1 Activation. FEBS J (2022). doi: 10.1111/febs.16375 PMC9309721 35080342

[147] Dai S Tang Z Cao J Zhou W Li H Sampson S. Suppression of the HSF1-Mediated Proteotoxic Stress Response by the Metabolic Stress Sensor AMPK. EMBO J (2015) 34(3):275–93. doi: 10.15252/embj.201489062 PMC4339117 25425574

[148] Kim S-A Yoon J-H Lee S-H Ahn S-G. Polo-Like Kinase 1 Phosphorylates Heat Shock Transcription Factor 1 and Mediates Its Nuclear Translocation During Heat Stress*. J Biol Chem (2005) 280(13):12653–7. doi: 10.1074/jbc.M411908200 15661742

[149] Lee Y-J Kim E-H Lee JS Jeoung D Bae S Kwon SH. HSF1 as a Mitotic Regulator: Phosphorylation of HSF1 by Plk1 Is Essential for Mitotic Progression. Cancer Res (2008) 68(18):7550–60. doi: 10.1158/0008-5472.CAN-08-0129 18794143

[150] Budzyński MA Puustinen MC Joutsen J Sistonen L. Uncoupling Stress-Inducible Phosphorylation of Heat Shock Factor 1 From Its Activation. Mol Cell Biol (2015) 35(14):2530–40. doi: 10.1128/MCB.00816-14 PMC4475925 25963659

[151] Krakowiak J Zheng X Patel N Feder ZA Anandhakumar J Valerius K. Hsf1 and Hsp70 Constitute a Two-Component Feedback Loop That Regulates the Yeast Heat Shock Response. eLife (2018) 7:e31668. doi: 10.7554/eLife.31668 29393852 PMC5809143

[152] Zheng X Krakowiak J Patel N Beyzavi A Ezike J Khalil AS. Dynamic Control of Hsf1 During Heat Shock by a Chaperone Switch and Phosphorylation. eLife (2016) 5:e18638. doi: 10.7554/eLife.18638 27831465 PMC5127643

[153] Raychaudhuri S Loew C Körner R Pinkert S Theis M Hayer-Hartl M. Interplay of Acetyltransferase EP300 and the Proteasome System in Regulating Heat Shock Transcription Factor 1. Cell (2014) 156(5):975–85. doi: 10.1016/j.cell.2014.01.055 24581496

[154] Westerheide SD Anckar J Stevens SMJ Sistonen L Morimoto RI. Stress-Inducible Regulation of Heat Shock Factor 1 by the Deacetylase SIRT1. Sci (New York NY) (2009) 323(5917):1063–6. doi: 10.1126/science.1165946 PMC3429349 19229036

[155] Raynes R Pombier KM Nguyen K Brunquell J Mendez JE Westerheide SD. The SIRT1 Modulators AROS and DBC1 Regulate HSF1 Activity and the Heat Shock Response. PLoS One (2013) 8(1):e54364. doi: 10.1371/journal.pone.0054364 23349863 PMC3548779

[156] Geng H Liu Q Xue C David LL Beer TM Thomas GV. Hif1α Protein Stability Is Increased by Acetylation at Lysine 709*. J Biol Chem (2012) 287(42):35496–505. doi: 10.1074/jbc.M112.400697 PMC3471753 22908229

[157] Laemmle A Lechleiter A Roh V Schwarz C Portmann S Furer C. Inhibition of SIRT1 Impairs the Accumulation and Transcriptional Activity of HIF-1α Protein Under Hypoxic Conditions. PLoS One (2012) 7(3):1–12. doi: 10.1371/journal.pone.0033433 PMC3316573 22479397

[158] Zelin E Freeman BC. Lysine Deacetylases Regulate the Heat Shock Response Including the Age-Associated Impairment of HSF1. J Mol Biol (2015) 427(7):1644–54. doi: 10.1016/j.jmb.2015.02.010 PMC4357550 25688804

[159] Ceccacci E Minucci S. Inhibition of Histone Deacetylases in Cancer Therapy: Lessons From Leukaemia. Br J Cancer (2016) 114(6):605–11. doi: 10.1038/bjc.2016.36 PMC4800301 26908329

[160] Kmiecik SW Drzewicka K Melchior F Mayer MP. Heat Shock Transcription Factor 1 is SUMOylated in the Activated Trimeric State. J Biol Chem (2021) 296:100324. doi: 10.1016/j.jbc.2021.100324 33493517 PMC7949154

[161] Brunet Simioni M de Thonel A Hammann A Joly AL Bossis G Fourmaux E. Heat Shock Protein 27 is Involved in SUMO-2/3 Modification of Heat Shock Factor 1 and Thereby Modulates the Transcription Factor Activity. Oncogene (2009) 28(37):3332–44. doi: 10.1038/onc.2009.188 19597476

[162] Anckar J Hietakangas V Denessiouk K Thiele DJ Johnson MS Sistonen L. Inhibition of DNA Binding by Differential Sumoylation of Heat Shock Factors. Mol Cell Biol (2006) 26(3):955–64. doi: 10.1128/MCB.26.3.955-964.2006 PMC1347039 16428449

[163] Lecomte S Reverdy L le Quément C le Masson F Amon A le Goff P. Unraveling Complex Interplay Between Heat Shock Factor 1 and 2 Splicing Isoforms. PLoS One (2013) 8(2):e56085. doi: 10.1371/journal.pone.0056085 23418516 PMC3572029

[164] Barna J Csermely P Vellai T. Roles of Heat Shock Factor 1 Beyond the Heat Shock Response. Cell Mol Life Sci (2018) 75(16):2897–916. doi: 10.1007/s00018-018-2836-6 PMC11105406 29774376

[165] Cerami E Gao J Dogrusoz U Gross BE Sumer SO Aksoy BA. The Cbio Cancer Genomics Portal: An Open Platform for Exploring Multidimensional Cancer Genomics Data. Cancer Discovery (2012) 2(5):401. doi: 10.1158/2159-8290.CD-12-0095 22588877 PMC3956037

[166] Gao J Aksoy BA Dogrusoz U Dresdner G Gross B Sumer SO. Integrative Analysis of Complex Cancer Genomics and Clinical Profiles Using the Cbioportal. Sci Signaling (2013) 6(269):pl1–1. doi: 10.1126/scisignal.2004088 PMC4160307 23550210

[167] Wan T Shao J Hu B Liu G Luo P Zhou Y. Prognostic Role of HSF1 Overexpression in Solid Tumors: A Pooled Analysis of 3,159 Patients. OncoTargets Ther (2018) 11:383–93. doi: 10.2147/OTT.S153682 PMC5775745 29398920

[168] Mendillo ML Santagata S Koeva M Bell GW Hu R Tamimi RM. HSF1 Drives a Transcriptional Program Distinct From Heat Shock to Support Highly Malignant Human Cancers. Cell (2012) 150(3):549–62. doi: 10.1016/j.cell.2012.06.031 PMC3438889 22863008

[169] Wang Q Zhang Y-C Zhu L-F Pan L Yu M Shen W-L. Heat Shock Factor 1 in Cancer-Associated Fibroblasts is a Potential Prognostic Factor and Drives Progression of Oral Squamous Cell Carcinoma. Cancer Sci (2019) 110(5):1790–803. doi: 10.1111/cas.13991 PMC6501034 30843645

[170] Dai W Ye J Zhang Z Yang L Ren H Wu H. Increased Expression of Heat Shock Factor 1 (HSF1) is Associated With Poor Survival in Gastric Cancer Patients. Diagn Pathol (2018) 13(1):80. doi: 10.1186/s13000-018-0755-3 30326922 PMC6191912

[171] Tsukao Y Yamasaki M Miyazaki Y Makino T Takahashi T Kurokawa Y. Overexpression of Heat-Shock Factor 1 is Associated With a Poor Prognosis in Esophageal Squamous Cell Carcinoma. Oncol Lett (2017) 13(3):1819–25. doi: 10.3892/ol.2017.5637 PMC5403275 28454329

[172] Salnikow K Zhitkovich A. Genetic and Epigenetic Mechanisms in Metal Carcinogenesis and Cocarcinogenesis: Nickel, Arsenic, and Chromium. Chem Res Toxicol (2008) 21(1):28–44. doi: 10.1021/tx700198a 17970581 PMC2602826

[173] Ortega-Atienza S Rubis B McCarthy C Zhitkovich A. Formaldehyde Is a Potent Proteotoxic Stressor Causing Rapid Heat Shock Transcription Factor 1 Activation and Lys48-Linked Polyubiquitination of Proteins. Am J Pathol (2016) 186(11):2857–68. doi: 10.1016/j.ajpath.2016.06.022 PMC5222959 27639166

[174] Brusselaers N Ekwall K Durand-Dubief M. Copy Number of 8q24.3 Drives HSF1 Expression and Patient Outcome in Cancer: An Individual Patient Data Meta-Analysis. Hum Genomics (2019) 13(1):54. doi: 10.1186/s40246-019-0241-3 31699156 PMC6836670

[175] Zhang CQ Williams H Prince TL Ho ES. Overexpressed HSF1 Cancer Signature Genes Cluster in Human Chromosome 8q. Hum Genomics (2017) 11(1):35. doi: 10.1186/s40246-017-0131-5 29268782 PMC5740759

[176] Gökmen-Polar Y Badve S. Upregulation of HSF1 in Estrogen Receptor Positive Breast Cancer. Oncotarget (2016) 7(51):84239–45. doi: 10.18632/oncotarget.12438 PMC5356658 27713164

[177] Chen F Fan Y Cao P Liu B Hou J Zhang B. Pan-Cancer Analysis of the Prognostic and Immunological Role of HSF1: A Potential Target for Survival and Immunotherapy. Oxid Med Cell Longevity (2021) 2021:5551036. doi: 10.1155/2021/5551036 PMC8238600 34239690

[178] Santagata S Hu R Lin NU Mendillo ML Collins LC Hankinson SE. High Levels of Nuclear Heat-Shock Factor 1 (HSF1) are Associated With Poor Prognosis in Breast Cancer. Proc Natl Acad Sci USA (2011) 108(45):18378–83. doi: 10.1073/pnas.1115031108 PMC3215027 22042860

[179] Vydra N Janus P Toma-Jonik A Stokowy T Mrowiec K Korfanty J. 17beta-Estradiol Activates HSF1 *via* MAPK Signaling in ERalpha-Positive Breast Cancer Cells. Cancers (2019) 11(10):1533. doi: 10.3390/cancers11101533 PMC6826487 31614463

[180] Khaleque MA Bharti A Gong J Gray PJ Sachdev V Ciocca DR. Heat Shock Factor 1 Represses Estrogen-Dependent Transcription Through Association With MTA1. Oncogene (2008) 27(13):1886–93. doi: 10.1038/sj.onc.1210834 17922035

[181] Silveira MA Tav C Bérube-Simard F-A Cuppens T Leclercq M Fournier É. Modulating HSF1 Levels Impacts Expression of the Estrogen Receptor α and Antiestrogen Response. Life Sci Alliance (2021) 4(5):e202000811. doi: 10.26508/lsa.202000811 33593922 PMC7893817

[182] Hoter A Naim HY. Heat Shock Proteins and Ovarian Cancer: Important Roles and Therapeutic Opportunities. Cancers (2019) 11(9):1389. doi: 10.3390/cancers11091389 PMC6769485 31540420

[183] Meng L Gabai VL Sherman MY. Heat-Shock Transcription Factor HSF1 has a Critical Role in Human Epidermal Growth Factor Receptor-2-Induced Cellular Transformation and Tumorigenesis. Oncogene (2010) 29(37):5204–13. doi: 10.1038/onc.2010.277 PMC2940982 20622894

[184] Xi C Hu Y Buckhaults P Moskophidis D Mivechi NF. Heat Shock Factor Hsf1 Cooperates With ErbB2 (Her2/Neu) Protein to Promote Mammary Tumorigenesis and Metastasis. J Biol Chem (2012) 287(42):35646–57. doi: 10.1074/jbc.M112.377481 PMC3471706 22847003

[185] Schulz R Streller F Scheel AH Ruschoff J Reinert M-C Dobbelstein M. HER2/ErbB2 Activates HSF1 and Thereby Controls HSP90 Clients Including MIF in HER2-Overexpressing Breast Cancer. Cell Death Dis (2014) 5:e980. doi: 10.1038/cddis.2013.508 24384723 PMC4040658

[186] Yallowitz A Ghaleb A Garcia L Alexandrova EM Marchenko N. Heat Shock Factor 1 Confers Resistance to Lapatinib in ERBB2-Positive Breast Cancer Cells. Cell Death Dis (2018) 9(6):621. doi: 10.1038/s41419-018-0691-x 29799521 PMC5967334

[187] Krzyzanowski PM Sircoulomb F Yousif F Normand J La Rose J E Francis K. Regional Perturbation of Gene Transcription is Associated With Intrachromosomal Rearrangements and Gene Fusion Transcripts in High Grade Ovarian Cancer. Sci Rep (2019) 9(1):3590. doi: 10.1038/s41598-019-39878-9 30837567 PMC6401071

[188] Coetzee SG Shen HC Hazelett DJ Lawrenson K Kuchenbaecker K Tyrer J. Cell-Type-Specific Enrichment of Risk-Associated Regulatory Elements at Ovarian Cancer Susceptibility Loci. Hum Mol Genet (2015) 24(13):3595–607. doi: 10.1093/hmg/ddv101 PMC4459387 25804953

[189] Chen Y-F Dong Z Xia Y Tang J Peng L Wang S. Nucleoside Analog Inhibits microRNA-214 Through Targeting Heat-Shock Factor 1 in Human Epithelial Ovarian Cancer. Cancer Sci (2013) 104(12):1683–9. doi: 10.1111/cas.12277 PMC7653516 24033540

[190] Chen Y-F Wang S-Y Yang Y-H Zheng J Liu T Wang L. Targeting HSF1 Leads to an Antitumor Effect in Human Epithelial Ovarian Cancer. Int J Mol Med (2017) 39(6):1564–70. doi: 10.3892/ijmm.2017.2978 28487934

[191] Yasuda K Hirohashi Y Mariya T Murai A Tabuchi Y Kuroda T. Phosphorylation of HSF1 at Serine 326 Residue is Related to the Maintenance of Gynecologic Cancer Stem Cells Through Expression of HSP27. Oncotarget (2017) 8(19):31540–53. doi: 10.18632/oncotarget.16361 PMC5458228 28415561

[192] Powell CD Paullin TR Aoisa C Menzie CJ Ubaldini A Westerheide SD. The Heat Shock Transcription Factor HSF1 Induces Ovarian Cancer Epithelial-Mesenchymal Transition in a 3D Spheroid Growth Model. PLoS One (2016) 11(12):e0168389. doi: 10.1371/journal.pone.0168389 27997575 PMC5172610

[193] Wilson AL Moffitt LR Duffield N Rainczuk A Jobling TW Plebanski M. Autoantibodies Against HSF1 and CCDC155 as Biomarkers of Early-Stage, High-Grade Serous Ovarian Cancer. Cancer Epidemiol Prev Biomarkers (2018) 27(2):183–92. doi: 10.1158/1055-9965.EPI-17-0752 29141850

[194] Sieh W Köbel M Longacre TA Bowtell DD deFazio A Goodman MT. Hormone-Receptor Expression and Ovarian Cancer Survival: An Ovarian Tumor Tissue Analysis Consortium Study. Lancet Oncol (2013) 14(9):853–62. doi: 10.1016/S1470-2045(13)70253-5 PMC4006367 23845225

[195] Cui J Tian H Chen G. Upregulation of Nuclear Heat Shock Factor 1 Contributes to Tumor Angiogenesis and Poor Survival in Patients With Non-Small Cell Lung Cancer. Ann Thorac Surgery (2015) 100(2):465–72. doi: 10.1016/j.athoracsur.2015.03.021 26095102

[196] Carter JD. Heat Shock Transcription Factor 1 (HSF1) Is a Novel Supporter of NSCLC Anoikis Resistance Independent of Heat Shock Proteins. Philadelphia:University of the Sciences in Philadelphia (2017).

[197] Hoj JP Mayro B Pendergast AM. The ABL2 Kinase Regulates an HSF1-Dependent Transcriptional Program Required for Lung Adenocarcinoma Brain Metastasis. Proc Natl Acad Sci USA (2020) 117(52):33486–95. doi: 10.1073/pnas.2007991117 PMC7777191 33318173

[198] Scherz-Shouval R Santagata S Mendillo ML Sholl LM Ben-Aharon I Beck AH. The Reprogramming of Tumor Stroma by HSF1 Is a Potent Enabler of Malignancy. Cell (2014) 158(3):564–78. doi: 10.1016/j.cell.2014.05.045 PMC4249939 25083868

[199] Lee S Jung J Lee Y-J Kim S-K Kim J-A Kim B-K. Targeting HSF1 as a Therapeutic Strategy for Multiple Mechanisms of EGFR Inhibitor Resistance in EGFR Mutant Non-Small-Cell Lung Cancer. Cancers (2021) 13(12):2987. doi: 10.3390/cancers13122987 34203709 PMC8232331

[200] Yoon T Kang G-Y Han A-R Seo E-K Lee Y-S. 2,4-Bis(4-Hydroxybenzyl)Phenol Inhibits Heat Shock Transcription Factor 1 and Sensitizes Lung Cancer Cells to Conventional Anticancer Modalities. J Natural Products (2014) 77(5):1123–9. doi: 10.1021/np4009333 24746225

[201] Cigliano A Wang C Pilo MG Szydlowska M Brozzetti S Latte G. Inhibition of HSF1 Suppresses the Growth of Hepatocarcinoma Cell Lines *In Vitro* and AKT-Driven Hepatocarcinogenesis in Mice. Oncotarget (2017) 8(33):54149–59. doi: 10.18632/oncotarget.16927 PMC5589569 28903330

[202] Liu P Ge M Hu J Li X Che L Sun K. A Functional Mammalian Target of Rapamycin Complex 1 Signaling is Indispensable for C-Myc-Driven Hepatocarcinogenesis. Hepatol (Baltimore Md) (2017) 66(1):167–81. doi: 10.1002/hep.29183 PMC5481473 28370287

[203] Cigliano A Pilo MG Li L Latte G Szydlowska M Simile MM. Deregulated C-Myc Requires a Functional HSF1 for Experimental and Human Hepatocarcinogenesis. Oncotarget (2017) 8(53):90638–50. doi: 10.18632/oncotarget.21469 PMC5710874 29207593

[204] Cen H Zheng S Fang Y-M Tang X-P Dong Q. Induction of HSF1 Expression is Associated With Sporadic Colorectal Cancer. World J Gastroenterol (2004) 10(21):3122–6. doi: 10.3748/wjg.v10.i21.3122 PMC4611254 15457556

[205] Li J Song P Jiang T Dai D Wang H Sun J. Heat Shock Factor 1 Epigenetically Stimulates Glutaminase-1-Dependent mTOR Activation to Promote Colorectal Carcinogenesis. Mol Therapy (2018) 26(7):1828–39. doi: 10.1016/j.ymthe.2018.04.014 PMC6035735 29730197

[206] Levi-Galibov O Lavon H Wassermann-Dozorets R Pevsner-Fischer M Mayer S Wershof E. Heat Shock Factor 1-Dependent Extracellular Matrix Remodeling Mediates the Transition From Chronic Intestinal Inflammation to Colon Cancer. Nat Commun (2020) 11(1):6245. doi: 10.1038/s41467-020-20054-x 33288768 PMC7721883

[207] Kourtis N Moubarak RS Aranda-Orgilles B Lui K Aydin IT Trimarchi T. FBXW7 Modulates Cellular Stress Response and Metastatic Potential Through ​HSF1 Post-Translational Modification. Nat Cell Biol (2015) 17(3):322–32. doi: 10.1038/ncb3121 PMC4401662 25720964

[208] Nakamura Y Fujimoto M Fukushima S Nakamura A Hayashida N Takii R. Heat Shock Factor 1 is Required for Migration and Invasion of Human Melanoma In Vitro and *In Vivo* . Cancer Lett (2014) 354(2):329–35. doi: 10.1016/j.canlet.2014.08.029 25194503

[209] Daniels GA Sanchez-Perez L Diaz RM Kottke T Thompson J Lai M. A Simple Method to Cure Established Tumors by Inflammatory Killing of Normal Cells. Nat Biotechnol (2004) 22(9):1125–32. doi: 10.1038/nbt1007 15300260

[210] Taha EA Ono K Eguchi T. Roles of Extracellular HSPs as Biomarkers in Immune Surveillance and Immune Evasion. Int J Mol Sci (2019) 20(18):4588. doi: 10.3390/ijms20184588 PMC6770223 31533245

[211] Haslam A Prasad V. Estimation of the Percentage of US Patients With Cancer Who Are Eligible for and Respond to Checkpoint Inhibitor Immunotherapy Drugs. JAMA Netw Open (2019) 2(5):e192535. doi: 10.1001/jamanetworkopen.2019.2535 31050774 PMC6503493

[212] Seliger B. Basis of PD1/PD-L1 Therapies. J Clin Med (2019) 8(12):2168. doi: 10.3390/jcm8122168 PMC6947170 31817953

[213] Yang T Ren C Lu C Qiao P Han X Wang L. Phosphorylation of HSF1 by PIM2 Induces PD-L1 Expression and Promotes Tumor Growth in Breast Cancer. Cancer Res (2019) 79(20):5233–44. doi: 10.1158/0008-5472.CAN-19-0063 31409638

[214] Chatterjee S Chakraborty P Daenthanasanmak A Iamsawat S Andrejeva G Luevano LA. Targeting PIM Kinase With PD1 Inhibition Improves Immunotherapeutic Antitumor T-Cell Response. Clin Cancer Res (2019) 25(3):1036–49. doi: 10.1158/1078-0432.CCR-18-0706 PMC6361669 30327305

[215] Li H Sui X Wang Z Fu H Wang Z Yuan M. A New Antisarcoma Strategy: Multisubtype Heat Shock Protein/Peptide Immunotherapy Combined With PD-L1 Immunological Checkpoint Inhibitors. Clin Trans Oncol (2021) 23(8):1688–704. doi: 10.1007/s12094-021-02570-4 PMC8238772 33792840

[216] Jaeger AM Stopfer L Lee S Gaglia G Sandel D Santagata S. Rebalancing Protein Homeostasis Enhances Tumor Antigen Presentation. Clin Cancer Res (2019) 25(21):6392–405. doi: 10.1158/1078-0432.CCR-19-0596 PMC6825536 31213460

[217] Ibrahim EC Morange M Dausset J Carosella ED Paul P. Heat Shock and Arsenite Induce Expression of the Nonclassical Class I Histocompatibility HLA-G Gene in Tumor Cell Lines. Cell Stress Chaperones (2000) 5(3):207–18. doi: 10.1379/1466-1268(2000)005<0207:HSAAIE>2.0.CO;2 PMC312887 11005379

[218] Amodio G Gregori S. HLA-G Genotype/Expression/Disease Association Studies: Success, Hurdles, and Perspectives. Front Immunol (2020) 11:1178. doi: 10.3389/fimmu.2020.01178 32733439 PMC7360675

[219] Morandi F Rizzo R Fainardi E Rouas-Freiss N Pistoia V. Recent Advances in Our Understanding of HLA-G Biology: Lessons From a Wide Spectrum of Human Diseases. J Immunol Res (2016) 2016:4326495. doi: 10.1155/2016/4326495 27652273 PMC5019910

[220] Schilling D Kühnel A Tetzlaff F Konrad S Multhoff G. NZ28-Induced Inhibition of HSF1, SP1 and NF-κb Triggers the Loss of the Natural Killer Cell-Activating Ligands MICA/B on Human Tumor Cells. Cancer Immunol Immunother (2015) 64(5):599–608. doi: 10.1007/s00262-015-1665-9 25854583 PMC4412431

[221] Nikolaou M Pavlopoulou A Georgakilas AG Kyrodimos E. The Challenge of Drug Resistance in Cancer Treatment: A Current Overview. Clin Exp Metastasis (2018) 35(4):309–18. doi: 10.1007/s10585-018-9903-0 29799080

[222] Kioka N Yamano Y Komano T Ueda K. Heat-Shock Responsive Elements in the Induction of the Multidrug Resistance Gene (MDR1). FEBS Lett (1992) 301(1):37–40. doi: 10.1016/0014-5793(92)80205-U 1360409

[223] Vilaboa NE Galan A Troyano A de Blas E Aller P. Regulation of Multidrug Resistance 1 (MDR1)/P-Glycoprotein Gene Expression and Activity by Heat-Shock Transcription Factor 1 (HSF1). J Biol Chem (2000) 275(32):24970–6. doi: 10.1074/jbc.M909136199 10816597

[224] Kim SH Yeo GS Lim YS Kang CD Kim CM Chung BS. Suppression of Multidrug Resistance *via* Inhibition of Heat Shock Factor by Quercetin in MDR Cells. Exp Mol Med (1998) 30(2):87–92. doi: 10.1038/emm.1998.13 9873828

[225] Tchénio T Havard M Martinez LA Dautry F. Heat Shock-Independent Induction of Multidrug Resistance by Heat Shock Factor 1. Mol Cell Biol (2006) 26(2):580–91. doi: 10.1128/MCB.26.2.580-591.2006 PMC1346900 16382149

[226] Vydra N Toma A Glowala-Kosinska M Gogler-Piglowska A Widlak W. Overexpression of Heat Shock Transcription Factor 1 Enhances the Resistance of Melanoma Cells to Doxorubicin and Paclitaxel. BMC Cancer (2013) 13:504. doi: 10.1186/1471-2407-13-504 24165036 PMC4231344

[227] Dokladny K Zuhl MN Mandell M Bhattacharya D Schneider S Deretic V. Regulatory Coordination Between Two Major Intracellular Homeostatic Systems: Heat Shock Response and Autophagy. J Biol Chem (2013) 288(21):14959–72. doi: 10.1074/jbc.M113.462408 PMC3663517 23576438

[228] Luo T Fu J Xu A Su B Ren Y Li N. PSMD10/gankyrin Induces Autophagy to Promote Tumor Progression Through Cytoplasmic Interaction With ATG7 and Nuclear Transactivation of ATG7 Expression. Autophagy (2016) 12(8):1355–71. doi: 10.1080/15548627.2015.1034405 PMC4968225 25905985

[229] Desai S Liu Z Yao J Patel N Chen J Wu Y. Heat Shock Factor 1 (HSF1) Controls Chemoresistance and Autophagy Through Transcriptional Regulation of Autophagy-Related Protein 7 (ATG7). J Biol Chem (2013) 288(13):9165–76. doi: 10.1074/jbc.M112.422071 PMC3610989 23386620

[230] Zhang N Wu Y Lyu X Li B Yan X Xiong H. HSF1 Upregulates ATG4B Expression and Enhances Epirubicin-Induced Protective Autophagy in Hepatocellular Carcinoma Cells. Cancer Lett (2017) 409:81–90. doi: 10.1016/j.canlet.2017.08.039 28889000

[231] Luan Q Jin L Jiang CC Tay KH Lai F Liu XY. RIPK1 Regulates Survival of Human Melanoma Cells Upon Endoplasmic Reticulum Stress Through Autophagy. Autophagy (2015) 11(7):975–94. doi: 10.1080/15548627.2015.1049800 PMC4590596 26018731

[232] Parekh S. Targeting HSF1: A Prime Integrator of Proteotoxic Stress Response in Myeloma. Clin Cancer Res (2018) 24(10):2237–8. doi: 10.1158/1078-0432.CCR-18-0030 29440176

[233] Manasanch EE Orlowski RZ. Proteasome Inhibitors in Cancer Therapy. Nat Rev Clin Oncol (2017) 14(7):417–33. doi: 10.1038/nrclinonc.2016.206 PMC5828026 28117417

[234] Zaarur N Gabai VL Porco JA Calderwood S Sherman MY. Targeting Heat Shock Response to Sensitize Cancer Cells to Proteasome and Hsp90 Inhibitors. Cancer Res (2006) 66(3):1783–91. doi: 10.1158/0008-5472.CAN-05-3692 16452239

[235] Shah SP Nooka AK Jaye DL Bahlis NJ Lonial S Boise LH. Bortezomib-Induced Heat Shock Response Protects Multiple Myeloma Cells and Is Activated by Heat Shock Factor 1 Serine 326 Phosphorylation. Oncotarget (2016) 7(37):59727–41. doi: 10.18632/oncotarget.10847 PMC5312344 27487129

[236] Velayutham M Cardounel AJ Liu Z Ilangovan G. Discovering a Reliable Heat-Shock Factor-1 Inhibitor to Treat Human Cancers: Potential Opportunity for Phytochemists. Front Oncol (2018) 8:97. doi: 10.3389/fonc.2018.00097 29682483 PMC5897429

[237] Dong B Jaeger AM Thiele DJ. Inhibiting Heat Shock Factor 1 in Cancer: A Unique Therapeutic Opportunity. Trends Pharmacol Sci (2019) 40(12):986–1005. doi: 10.1016/j.tips.2019.10.008 31727393

[238] Yoon YJ Kim JA Shin KD Shin D-S Han YM Lee YJ. KRIBB11 Inhibits HSP70 Synthesis Through Inhibition of Heat Shock Factor 1 Function by Impairing the Recruitment of Positive Transcription Elongation Factor B to the Hsp70 Promoter. J Biol Chem (2011) 286(3):1737–47. doi: 10.1074/jbc.M110.179440 PMC3023468 21078672

[239] Vilaboa N Bore A Martin-Saavedra F Bayford M Winfield N Firth-Clark S. New Inhibitor Targeting Human Transcription Factor HSF1: Effects on the Heat Shock Response and Tumor Cell Survival. Nucleic Acids Res (2017) 45(10):5797–817. doi: 10.1093/nar/gkx194 PMC5449623 28369544

[240] Salamanca HH Antonyak MA Cerione RA Shi H Lis JT. Inhibiting Heat Shock Factor 1 in Human Cancer Cells With a Potent RNA Aptamer. PLoS One (2014) 9(5):e96330. doi: 10.1371/journal.pone.0096330 24800749 PMC4011729

[241] Chen B-C Tu S-L Zheng B-A Dong Q-J Wan Z-A Dai Q-Q. Schizandrin A Exhibits Potent Anticancer Activity in Colorectal Cancer Cells by Inhibiting Heat Shock Factor 1. Biosci Rep (2020) 40(3):BSR20200203. doi: 10.1042/BSR20200203 32110802 PMC7069920

